# The role of signaling crosstalk of microglia in hippocampus on progression of ageing and Alzheimer's disease

**DOI:** 10.1016/j.jpha.2023.05.008

**Published:** 2023-05-15

**Authors:** He Li, Tianyuan Ye, Xingyang Liu, Rui Guo, Xiuzhao Yang, Yangyi Li, Dongmei Qi, Yihua Wei, Yifan Zhu, Lei Wen, Xiaorui Cheng

**Affiliations:** a Innovative Institute of Chinese Medicine and Pharmacy, Shandong University of Traditional Chinese Medicine, Jinan, 250355, China; b Xiamen Key Laboratory for TCM Dampness Disease, Neurology & Immunology Research, Department of Traditional Chinese Medicine, Xiang'an Hospital, School of Medicine, Xiamen University, Xiamen, Fujian, 361102, China; c Experimental Center, Shandong University of Traditional Chinese Medicine, Jinan, 250355, China

**Keywords:** Alzheimer's disease, Microglia, Immune, Crosstalk, Chemokine, Colony-stimulating factor

## Abstract

Based on single-cell sequencing of the hippocampi of 5× familiar Alzheimer's disease (5× FAD) and wild type mice at 2-, 12-, and 24-month of age, we found an increased percentage of microglia in aging and Alzheimer's disease (AD) mice. Blood brain barrier injury may also have contributed to this increase. Immune regulation by microglia plays a major role in the progression of aging and AD, according to the functions of 41 intersecting differentially expressed genes in microglia. Signaling crosstalk between C−C motif chemokine ligand (CCL) and major histocompatibility complex-1 bridges intercellular communication in the hippocampus during aging and AD. The amyloid precursor protein (APP) and colony stimulating factor (CSF) signals drive 5× FAD to deviate from aging track to AD occurrence among intercellular communication in hippocampus. Microglia are involved in the progression of aging and AD can be divided into 10 functional types. The strength of the interaction among microglial subtypes weakened with aging, and the CCL and CSF signaling pathways were the fundamental bridge of communication among microglial subtypes.

## Highlights

• BBB injury aggravates microglia increased and active.
• APP and CSF signals drive AD occurrence.
• M0 subtype in microglia may play a major role in the immune regulation.
• The interaction strength among microglial subtypes became weak with aging.
• CCL and CSF were the fundamental bridge of communication among microglia subtypes.

## Graphical abstract

**Figure:**
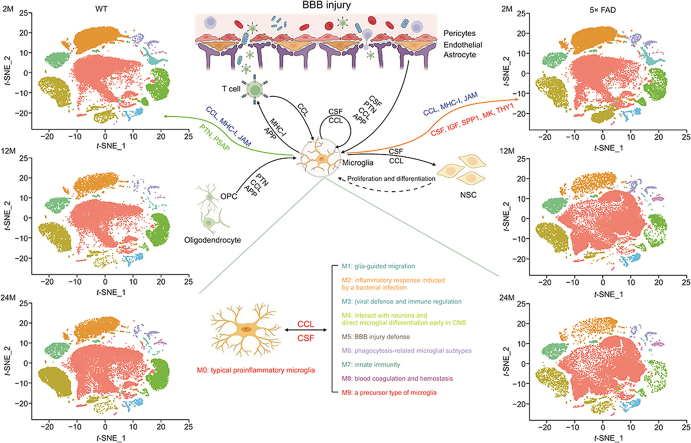

## 1 Introduction

Alzheimer's disease (AD) is a neurodegenerative disease characterized by cognitive decline that imposes a huge economic burden on society [1]. Aging, which is inevitable, is a primary risk factor for AD [2]. Delaying aging and preventing the emergence of dementia in older adults is the focus of the scientific community.

Plaques induced by amyloid beta protein (Aβ) and neurofibril tangles (NFT) induced by tau protein hyperphosphorylation are typical pathological products of AD [3,4]. Multiple clinicopathological associations have shown that Aβ and NFT are insufficient to adequately explain the extent of damage responses (e.g., loss of neurons and synapses) observed in AD brains, nor do they necessarily lead to cognitive impairment consequences [[5], [6], [7]]. A recent detailed examination of the brain with high-tangled load dementia (Braak V−VI) and elastic cognitive impairment showed that the most significant difference was the change in the glial cell response phenotype [7] and the expression profile of pro-inflammatory cytokines and microglial cell chemokines in dementia patients [8]. Glia-related neuroinflammation and innate immune dysregulation may lead to cognitive impairment.

Microglial-related neuroinflammation affects the trajectory of AD, and an individual's susceptibility to AD may depend in part on the behavioral phenotype of microglia [9]. The activated state of microglia in the brain of patients with AD is typically thought to be the polarization of the M1 or M2 phenotypes. Microglia differentiate between pro-inflammatory and anti-inflammatory functions based on changes in the expression of membrane receptors and secretory factors [10]. Analysis of the existing microglia transcriptome dataset from a mouse model of neuroinflammation and neurodegenerative disease showed that pro-inflammatory microglia appear earlier in the AD mouse model, characterized by pro-inflammatory genes, surface marker CD44, and potassium channel Kv1.3, whereas anti-inflammatory microglia express phagocytic genes, surface marker chemokine C−X−C motif receptor 4, and other regulatory factors [11]. Recent evidence indicates the complexity of microglial subtypes. Morphological and electrophysiological heterogeneity in the microglia associated with amyloid plaques has been observed in TgCRND8 mice [12]. However, using terms for macrophage polarization and M1 and M2 subtypes, this simplified model is not appropriate for describing microglia [10]. At present, research on the behavioral phenotypes of microglia is unclear.

Understanding the immune response patterns of microglia and the behavioral interaction mode of microglia with other cell types allows for the accurate targeting of microglia with impaired or abnormal responses, which has great potential for developing effective tools to delay aging and avoid neurodegenerative diseases.

Single-cell RNA sequencing (scRNA-seq) analysis can be used to comprehensively compare the distribution of cell transcriptomes in different samples and understand cell activity and functional status by integrating the analysis of epigenetic and biological functions and information communication. The scRNA-seq technique can accurately report the key phenotypic features of the immune subset [13].

In the present study, scRNA-seq analysis was performed on hippocampal samples from wide type (WT) and 5× familiar Alzheimer's disease (5× FAD) mice at 2-, 12-, and 24-month of age to map the clustering of hippocampal cells during aging and AD. We focused on the immune behavior and phenotypic characteristics of microglia. The outside and inside signal flow in the population of microglia reveals the crosstalk between neuroinflammatory pathways and cell behavior interactions guided by immune responses in the hippocampus. Our study found that blood-brain barrier (BBB) injury may increase the percentage of microglia during the progression of aging and AD. The signaling crosstalk of C−C motif chemokine ligand (CCL), major histocompatibility complex-1 (MHC-1), and colony stimulating factor (CSF) bridge intercellular communication in microglia with other cell types or subtypes of microglia in the hippocampus. Amyloid precursor protein (APP) and CSF signals drove 5× FAD mice to deviate from the aging track to AD occurrence.

## 2 Materials and methods

### 2.1 Animals

We procured 5× FAD mice (Jackson Laboratory, Bar Harbor, ME, USA) that carried the APP genes K670 N/M671L (Swedish), I716V (Florida), and V717I (London), and PS1 mutations M146L and L286V. Wild-type C57/BL6J mice served as normal controls. They were maintained in the specific pathogen free barrier environment at the Experimental Center of Shandong University of Traditional Chinese Medicine and Xiamen University in a controlled environment (room temperature 20−24 °C; humidity 45%–65%; and 12-h light/12-h dark cycle) and allowed free access to water and food. The experiment was approved by the Experimental Animal Welfare Ethics Review Committee of Shandong University of Traditional Chinese Medicine (Approve No.: SDUTCM20211025001). All operations followed the Animal Research: Reporting in Vivo Experiments (ARRIVE) guidelines. Female mice (2−24 months old) were used in the experiments. All the mice included in the experiment were genotyped. The primers used for genotyping were available as follows: PS1, 5′-AATAGAGAACGGCAGGAGCA-3’ (forward primer) and 5′-GCCATG AGGGCACTAATCAT-3’ (reverse primer); β-actin, 5′-CTAGGCCACAGAATTGAAAGATCT-3’ (forward primer) and 5′-GTAGGTGGAAATTCTAGCATCATCC-3’ (reverse primer). The primer synthesis was provided by Sangon Biotech (Shanghai) Co., Ltd. (Shanghai, China). A SteadyPure Universal Genomic DNA Extraction Kit (AG21009) provided by Accurate Biotechnology (Hunan) Co., Ltd. (Changsha, China) was used to extract DNA from mice. 2× Taq Master (P101−3) was obtained from ATG Biotechnology Co., Ltd. (Nanjing, China). Nucleic acid gel dye (TSJ003) and agarose (TSJ001) were purchased from Tsingke Biological Technology (Beijing, China). A Spark 2000 DNA Marker (AJ0101) purchased from Shandong Sparkjade Biotechnology Co., Ltd. (Shandong, China) was used to mark the molecular weight.

### 2.2 Preparation of hippocampus cell suspensions and libraries for single-cell RNA sequencing

The right hippocampi of 2-, 12-, and 24-month-old WT and 5× FAD mice were harvested for single-cell sequencing analyses. Each group contained three mice. The right hippocampus was rapidly stripped and 1× Dulbecco's phosphate-buffered saline (1× DPBS) washed. Bioyou® single cell sequencing tissue preservation solution (21903−10) provided from Shanghai Biotechnology Corporation (Shanghai, China) was used to maintain cell viability. The hippocampi were placed in a new centrifuge tube and a small digest was added to mince them into small pieces of approximately 1–2 mm^3^. The cut tissue was transferred to a 50 mL centrifuge tube with 5 mL of pancreatin solution (C3530; VivaCell, Shanghai, China), and shaken gently every 3−5 min. Later, the digests were passed through a pre-wetted cell strainer (70 μm, Log No.: 258365) by NEST Biotechnology Co., Ltd. (Wuxi, China), and the filtrate was collected in a new 50 mL centrifuge tube. The cell screen was washed with 10 mL of 1× DPBS (containing 2% fetal bovine serum), and the filtrate was collected in the same centrifuge tube. The supernatant was discarded after centrifugation, and the cells were resuspended and centrifuged. Cell counts and viability were calculated after lysing red blood cells and removing dead cells and trypan blue staining. The prepared cell suspension, 10x barcode gel beads, and oil were added to different chambers of the Chromium Chip G. Gel Beads-in-emulsion (GEM) was formed using the 10x Genomics Chromium System. GEM was reverse-transcribed using a polymerase chain reaction (PCR) machine, and a strand of complementary DNA (cDNA) was purified and enriched using magnetic beads. Next, cDNA amplification and inspection were performed. The cDNA concentration was determined using a Qubit, and the fragment size was determined using an Agilent 2100 Bioanalyzer. After cDNA amplification, enzyme slices were segmented, and magnetic beads were used to screen for optimal fragments. cDNA libraries containing the P5 and P7 adaptors were generated using end repair, tailing, and adaptor ligation of read2 sequencing primers, followed by PCR. Cluster generation and first-pass sequencing primer hybridization were completed following the Illumina User Guide, and the flow cell-carrying cluster was loaded. Paired-end sequencing was performed using the paired-end program. The sequencing process was controlled by the data collection software provided by Illumina, and real-time data analysis was performed. Shanghai Biotechnology Corporation provided single-cell sequencing services.

### 2.3 Single-cell RNA seq analysis

#### 2.3.1 Data quality control

Cell Ranger software was used to demultiplex the sequencing data based on 10× sample indices, and paired-end FASTQ files were generated. The filtered expression matrix output for each sample was read and processed using the R package Seurat V3. We calculated the unique molecular identifier and gene, mitochondrial, and ribosomal requirements for each sample to set a filtering threshold. The specific quality control standards are provided in Supplementary data 1. Sample information is provided in Supplementary data 2. Heterogeneity in the cell cycle phases drove substantial transcriptome variations that masked biological signals. The cell cycle in which each cell was located was determined by calculating the cell cycle score for each cell based on the expression of cell cycle genes [14]. This is necessary for evaluating the data quality, and the cell cycle effect in this experiment is weak and can be analyzed subsequently. Supplementary data 3 provides detailed cell cycle data.

#### 2.3.2 Multi-sample integration analysis and principal component analysis (PCA) dimensionality reduction

After quality control, 158533 cells from 18 samples were retained. Each sample included 10684, 12200, 13054, 12898, 11982, 12353, 3303, 10927, 7472, 11292, 10688, 12582, 8530, 10995, 11482, 9823, 6336, and 8239 cells, respectively. The median gene count is shown in Supplementary data 2. In the process of de-batching, 2000 highly variable genes were identified using *FindVariableFeatures* function from Seurat. The *FindVariableFeatures* function calculates the mean-variance result and selects genes with a larger variance in areas with different mean values. The specific dispersion value of each gene was calculated as follows: genes were divided into 20 interval classes based on the average expression of all genes, and the absolute value obtained after subtraction of the variance of the mean value of the gene in each interval and the variance of the median value was used as the normalized value of the dispersion of the group of genes. We selected the top 2000 genes with the largest variation based on the mean and variance of all genes in each sample for subsequent integration analysis. *Harmony* function was then used to remove the batch effect between the samples [15]. After processing the data using the LogNormalize and ScaleData algorithms, the *FindVariableFeature* function was applied to screen the feature genes for subsequent PCA. The first 30 PCAs were selected for follow-up analysis.

#### 2.3.3 Cell type annotation and marker genes analysis

Louvain was used for cluster analysis, and *t*-distributed Stochastic neighbor embedding (*t*-SNE) was used for dimension reduction [16,17]. To choose the best resolution, a cluster tree function was used, and a wide range of resolutions was tested. Based on manual annotation, 31 cell clusters were identified. We used *FindAllMarkers* function from Seurat to identify the marker genes of each cluster and the marker genes of all clusters were analyzed using the Wilcoxon algorithm and scored by group one versus the rest [18]. Genes that were highly expressed specifically for each cluster, with log (fold change) > 0.25, and expressed in at least 20% of the cells were selected as significant marker genes for the cluster (Supplementary data 4). According to classical markers and top gene annotations, the same cell types were merged to obtain ten cell types. Subsequently, we extracted the subsets of interest that were important to the analysis.

#### 2.3.4 Cell proportion analysis

The stacked bar chart shows the relative proportion of each cell type, which is equal to the number of cells in the target divided by the total number of cells. Student's *t*-test was used to evaluate the differences between the two groups of different strains at the same age. One-way analysis of variance followed by the Newman-Keuls comparison test was used to evaluate differences in the same strain at different ages.

#### 2.3.5 Differential gene screening and protein-protein interaction (PPI)

Wilcoxon's-sum rank test was used to test differential expression. Filter by Benjamini-Hochberg adjusted *P* < 0.05 and means fold difference of genes greater than 1.5. Intersections of differential genes were defined as common genes. We used STRING database (https://cn.string-db.org/) to predict protein-protein interaction networks. The combined score was used to evaluate the importance of the subnetwork molecules.

#### 2.3.6 Gene Ontology (GO) enrichment

In R software, the function “enrich GO” was used for GO enrichment analysis. Differences were set at a *q*-value <0.05.

#### 2.3.7 Cellular communication analysis

Cellchat (http://github.com/sqjin/CellChat) was used to infer, visualize, and analyze intercellular communication using scRNA-seq data [19]. We evaluated the communication between various cell types and investigated their signaling pathways.

### 2.4 Enzyme-linked immunospecific assay (ELISA)

Tissue lysate (abs9225) purchased from Absin Biotechnology Co., Ltd. (Shanghai, China) with 4-(2-aminoethyl)-benzenesulfonyl fluoride hydrochloride (HY-12821) purchased from MedChemExpress (Monmouth Junction, NJ, USA) were used to extract total protein. After the tissue was broken, it was centrifuged at 12000 r/min for 5 min at 4 °C. The supernatant was then subjected to bicinchoninic acid protein quantification (PC0020) which was provided by Beijing Solarbio Science & Technology Co., Ltd. (Beijing, China). Human amyloid beta 40 and 42 ELISA Kits (KHB3481, KHB3441) were obtained from Thermo Fisher Scientific Inc. (Vienna, Austria) according to the manufacturer's instructions.

### 2.5 Immunohistochemistry

Paraffin-embedded tissues were cut into 5 μM sections and placed in 60 °C oven for 2 h. The samples were soaked in xylene three times for 10 min each. Subsequently, 100%, 95%, and 75% (*V*/*V*) gradient alcohol solutions were used. Sodium citrate (AC28L123; Shanghai Life-iLab Biotech Co., Ltd., Shanghai, China) was placed in a water bath at 97 °C for 20 min. Immunostaining was performed using a blocking buffer (E674004; Sangon Biotech (Shanghai) Co., Ltd.) for 2 h. Incubation was done for 48 h after 500-fold dilution of the primary antibody (anti-Iba1, 019−19741; FUJIFILM Wako Pure Chemical Corporation, Tokyo, Japan) at 4 °C. Anti-rabbit IgG (A24221; Abbkine Scientific, Wuhan, China) was diluted 500-fold with immunostaining secondary antibody dilution buffer (E674005; Sangon Biotech (Shanghai) Co., Ltd.) and incubated for 2 h in the dark. Observations were made under the microscope after incubation with 4′,6-diamidino-2-phenylindole dihydrochloride (ID2250) obtained from Beijing Solarbio Science & Technology Co., Ltd. for 5 min.

## 3 Results

### 3.1 Single-cell sequencing identified the increased microglia percentage in the aging and AD

After data quality control, 0.4 resolution was used to perform the cell cluster. We obtained 31 hippocampal clusters (Fig. S1A) and identified their cell types using classical marker genes (Supplementary data 5). The top 3 genes of each cluster with high specificity are shown in a heatmap (Fig. S1B), which may be new cell markers for identifying cell types in the brain.

**Fig. 1:**
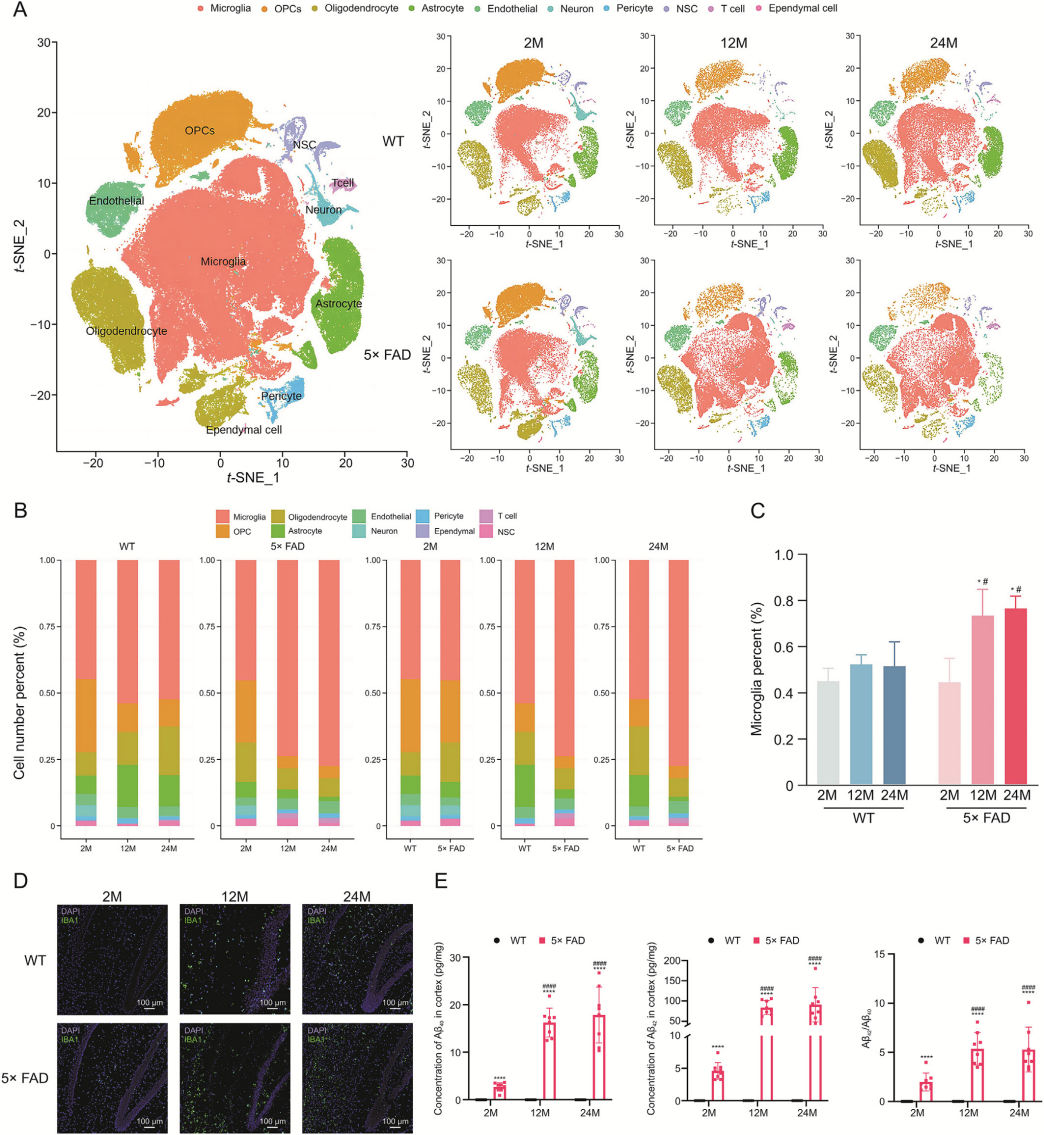
Microglia undergo significant changes with aging and Alzheimer's disease (AD). (A) *t*-Stochastic neighbor embedding (*t*-SNE) dimensional reduction and cell type interpretation of 158533 hippocampus cells from 2-, 12-, and 24-month-old wild type (WT) and 5× familiar Alzheimer's disease (5× FAD) mice (*n* = 3). (B) Stacked bar chart shows the relative proportion of each cell type in each biological group (*n* = 3 per group). (C) The scatter plot shows the percentage change in microglia in WT and 5× FAD mice at different ages. ^∗^*P* < 0.05 vs. the same strain at 2-month-old, one-way analysis of variance (ANOVA) followed by Newman-Keuls multiple comparison test; ^#^*P* < 0.05 vs. age-matched WT, Student's *t*-test, Graphpad 9.0.0 (*n* = 3). (D) Immunohistochemical verification of increased proportion of microglia. (E) Enzyme-linked immunospecific assay (ELISA) detection of Aβ_1-40_ and Aβ_1-42_. ^∗∗∗∗^*P* < 0.0001 vs. the same strain at 2-month-old, ANOVA followed by Newman-Keuls multiple comparison test; ^####^*P* < 0.0001 vs. age-matched WT, Student's *t*-test, Graphpad 9.0.0 (*n* = 3). OPCs: oligodendrocyte progenitor cells; NSC: neural stem cells; DAPI: 4’,6-diamidino-2-phenylindole.

The same cell types were combined to yield 10 cell types: microglia, oligodendrocyte progenitor cells (OPCs), oligodendrocytes, astrocytes, endothelial cells, neurons, pericytes, neural stem cells (NSC), T cells, and ependymal cells. We searched for representative genes of the merged cell types to serve as markers for cell type identification (Fig. S2). We determined the distribution of cells using *t*-SNE (Fig. 1A). As Fig. 1A shows, the cell types of the same strain changed as senescence occurred. At the age of 2 months, no significant differences were observed between WT and 5× FAD mice (Figs. 1A and B). However, there were obvious differences between the WT and 5× FAD mice at 12 and 24 months. The most prominent cell type was the microglia. In WT mice, microglial changes occur primarily at 12−24 months of age. In 5× FAD mice, microglia were significantly different at 2−12 months and were stable between 12 and 24 months (Fig. 1A).

We found that microglia occupied an important position in the cell number percentage (Fig. 1B). In WT mice, the proportion of microglia among all hippocampal cells was constant. However, in the 5× FAD mice, the proportion of microglia increased significantly from 2 to 12 months and 2 to 24 months of age. The proportion of microglia in the 5× FAD mice did not change from 12 to 24 months. Compared to age-matched WT mice, the proportion of microglia was significantly greater in 5× FAD mice at the ages of 12 and 24 months, and no obvious differences were observed at 2 months (Fig. 1C). This result was confirmed by immunohistochemistry. With aging and the progression of AD, microglia proliferate and become activated (Fig. 1D). These data suggested that premature aging and memory loss in 5× FAD mice were closely associated with microglial activation.

When using 5× FAD mice as classical AD mice mimicking Aβ pathology, Aβ deposition time, microglial proliferation, and activation time raise concerns. We tested Aβ_1−40_ and Aβ_1−42_ contents by ELISA (Fig. 1E). 5× FAD mice followed closely the initial rise in Aβ_1−42_ level observed at 1.5 months and showed the earliest amyloid deposition at 2 months and developed spatial memory deficits at 4−5 months of age [20]. Compared to 2-month-old 5× FAD mice, both 12- and 24-month-old had significantly higher Aβ_1−40_ and Aβ_1−42_; there was no significant difference in the deposition between the 12- and 24-month-old 5× FAD mice. This corresponded to the microglial ratio timeline. As for WT mice, the Aβ_1−40_ and Aβ_1−42_ were not detected as they were all lower than the lowest point of the detection line of the ELISA Kit.

### 3.2 BBB injury may increase microglia percentage in the progression of aging and AD

**Fig. 2:**
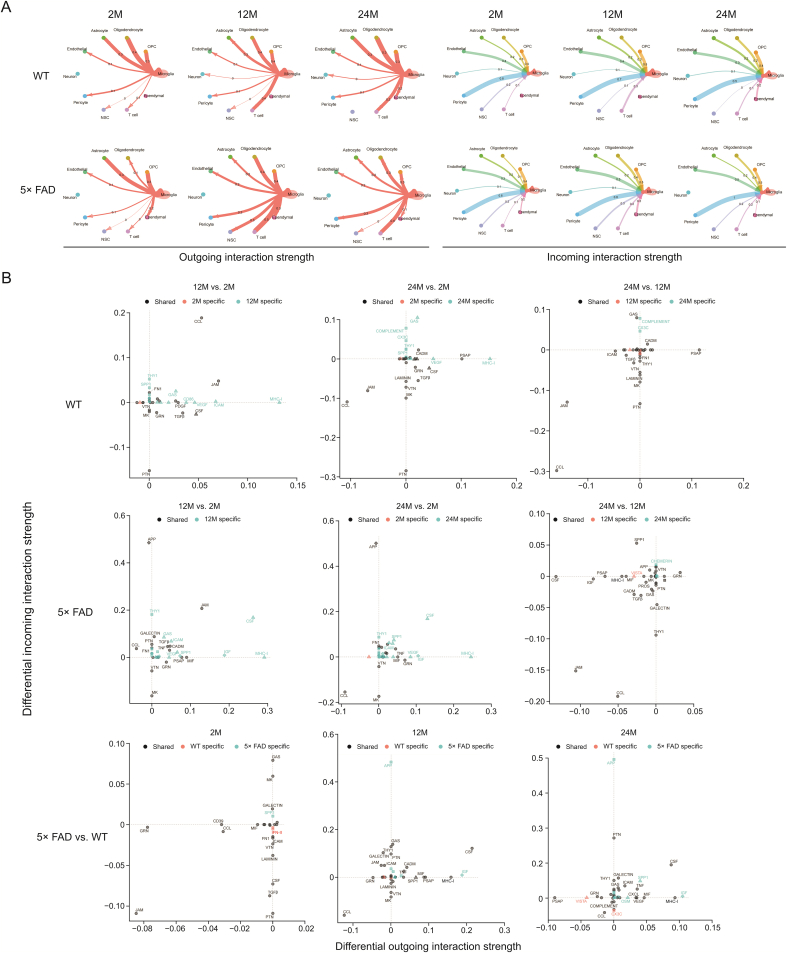
An atlas of microglia crosstalk with other types cell in hippocampus. (A) The circle diagram shows the interaction network of microglia with respect to the outgoing and incoming interaction strengths. The number on the line represents the communication strength. (B) Signaling changes of microglia in wild type (WT) and 5× familiar Alzheimer's disease (5× FAD) mice during different ages. Shape annotation: circle: shared; square: incoming specific; triangle: outgoing specific. Diamond: incoming and outgoing specific. OPC: oligodendrocyte progenitor cell; NSC: neural stem cell; CCL: C−C motif chemokine ligand; THY1: Thy-1 membrane glycoprotein; SPP1: phingosine-1-phosphate phosphatase 1; FN1: fibronectin; VTN: vitronectin; MK: midkine; GRN: progranulin; GAS: growth arrest-specific protein; PDGF: platelet-derived growth factor; TGFβ: transforming growth factor β; VEGF: vascular endothelial growth factor; CSF: colony-stimulating factor; JAM: junctional adhesion molecule; ICAM: intercellular adhesion molecule; MHC-I: major histocompatibility complex-1; PTN: pleiotrophin; CADM: cell adhesion molecule; PSAP: prosaposin; APP: amyloid precursor protein; MIF: macrophage migration inhibitory factor; CXCL: C−X−C motif chemokine ligand.

Signaling crosstalk between soluble and membrane-bound factors is critical for diverse cellular decisions [21,22]. To determine the potential cellular crosstalk involved in aging and AD, we used the CellChat package to calculate the cell communication probability of the scRNA-seq data. The results showed crosstalk between microglia and other cell types (Fig. 2A). Besides, the differential incoming and outgoing strengths were also analyzed (Fig. 2B). The interaction strength of microglial-astrocyte communication is relatively constant. Communication of microglia with the ependyma is unique in 5× FAD mice at 2- and 12-month of age. In addition, the communication of microglia with pericytes and endothelial cells was stronger in 5× FAD mice than in WT mice at 12- and 24-month of age. Endothelial cells are a component of the BBB [23], and disruption of the BBB is one of the factors that lead to inflammation in AD [24]. This suggests that the microglial immune response may affect the BBB function and integrity. CSF signaling regulates microglial proliferation during chronic neurodegeneration [25]. As an important growth factor, pleiotrophin (PTN) signaling promotes the development of the nervous system and stimulates cell proliferation and migration [26]. In the incoming microglial signal (Supplementary data 6), CSF signaling was mainly transmitted by pericytes and oligodendrocytes, and PTN signaling was mainly transmitted by pericytes, endothelial cells, astrocytes, OPCs, oligodendrocytes, and NSC. Peripheral endothelial and astrocyte-related BBB damage signals may promote abnormal microglial proliferation. Microglial communication with NSC in 5× FAD mice at 12- and 24-month-old is also stronger than that in WT mice, and the intensity is the highest at 12-month-old and is the main recipient of CSF signals, which may be related to the abnormal proliferation of microglia. NSC differentiate into microglia, which play an anti-inflammatory role [27]. The overall changes in signal communication for each cell type are shown in Fig. S3A. The differences in the strength of cell-cell communication between the same strain of mice of different ages and different strains of the same age were analyzed (Fig. S3B). In senescent 2-month-old WT and 5× FAD mice, the strength of cell-cell communication between OPCs and NSC was most significantly reduced in 12 and 24 months. NSC has the potential to differentiate into OPCs, a phenomenon that may be more pronounced in young mice. Between group comparisons for similar age groups revealed that 5× FAD mice had significantly higher incoming strengths of ependymal cells.

### 3.3 The immune regulation by microglia played a major role in the progression of aging and AD

**Fig. 3:**
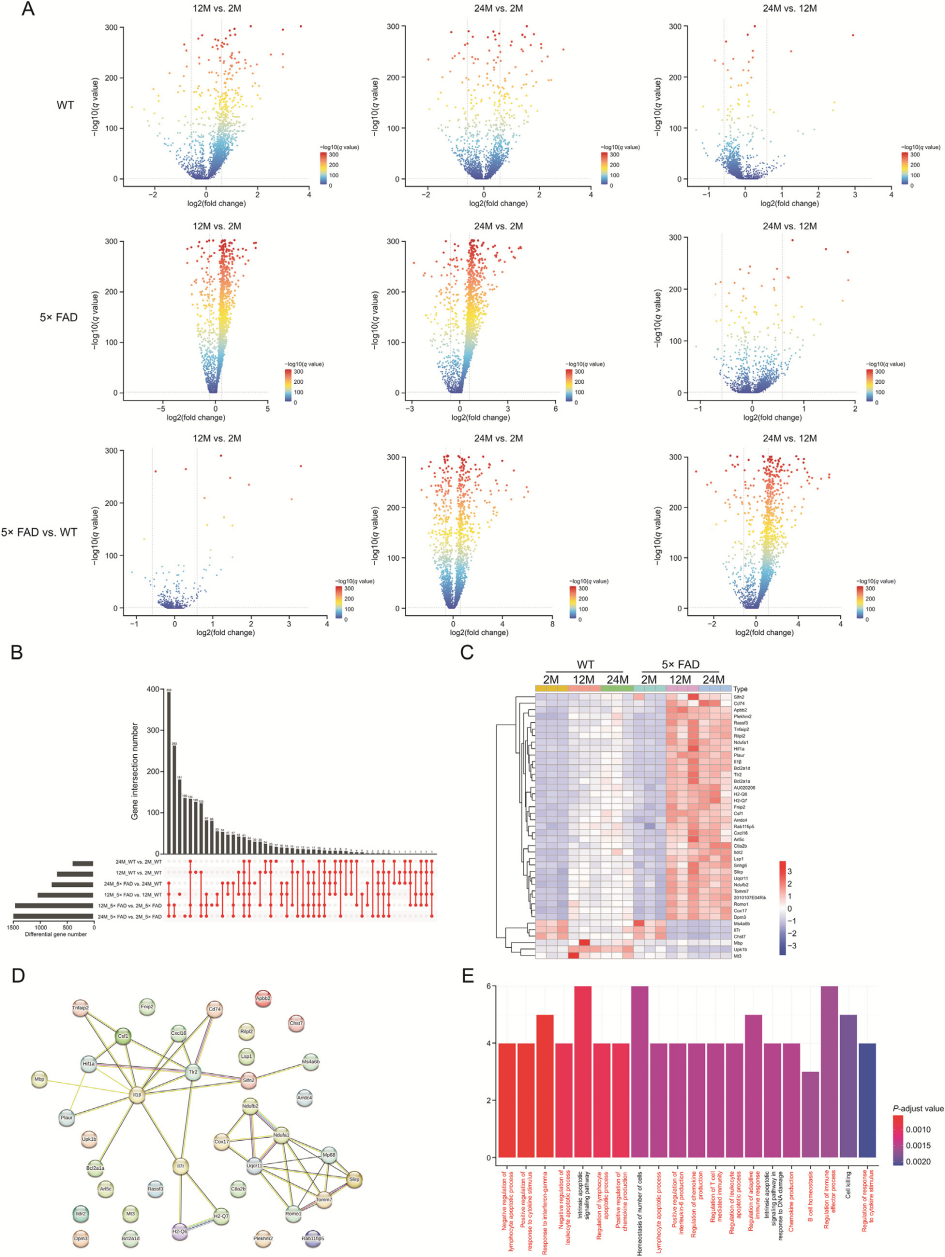
Microglia mainly play an immune role in aging and Alzheimer's disease. (A) The volcanic map shows the expression genes of microglia in wild type (WT) and 5× familiar Alzheimer's disease (5× FAD) mice with age. (B) Upset map shows 41 share genes can be obtained from 6 sets of intersections. The location of each red dot corresponds to the differential gene compared to the left. A red line has several points that illustrate that there are several gene sets taking the intersection. (C) Heatmap shows the expression of 41 share genes in each group. (D) Protein-protein interactions (PPIs) show the interaction of core protein and the minimum required interaction score equal to 0.4. (E) Gene Ontology (GO) analysis annotated the biological processes of common genes.

Because the changes in microglia were the most obvious, we focused on the changes in gene expression in microglia during aging and disease based on data from WT and 5× FAD mice. We compared the expression genes in microglia among the same strain at different ages and age-matched strains. The results showed that gene expression significantly changed from 2 to 12 months, 2 to 24 months, and 12 to 24 months in the microglia of WT or 5× FAD mice (Fig. 3A). From 12 to 24 months, there were fewer differential genetic changes in the microglia of WT or 5× FAD mice. This suggests that aging may be stable in microglia from middle to old age. Comparing WT and 5× FAD mice of the same age, there were few differentially expressed genes in microglia at 2 months of age, while many genes were differentially expressed at 12 and 24 months (Fig. 3A). This also coincided with the changing trend in the percentage of microglia (Figs. 1A−C). All changes and details are shown in Supplementary data 7.

Aging is a risk factor for AD [2]. We observed the intersection of differentially expressed genes in microglia between the aging process and AD to determine the core gene expression patterns of aging and AD. A total of 41 shared genes were found in the microglia of WT and 5× FAD mice of different ages (Fig. 3B). A heatmap was used to present the expression of the 41 genes (Fig. 3C). *Slfn2*, *Cd74*, and *Apbb2* expression increased, and *Ms4a6b*, *Il7r*, and *Chst7* expression decreased with age, especially in 5× FAD mice (Fig. 3C). Next, we mapped the PPI network of the core genes to observe their relationships (Fig. 3D). Immune-related *Tlr2* and *Il1β* have the most intensive relationship network. *Tlr2,* an innate immune receptor, plays an important role in the inflammatory processes and pathological development of AD [28]. And *Il1b*, a key mediator of inflammation, is highly expressed in the brain, particularly in the hippocampus has been confirmed [29]. Later we built subnetworks around *Tlr2* and *Il1β*, *Csf1* and *Cxcl1*6 had the highest combined score (Supplementary data 7). Pons et al. [30] found that conditional genetic deletion of CSF1 receptors in microglia ameliorated the pathophysiology of AD. *Cxcl16* levels are elevated in the CSF of cognitively impaired subjects [31]. However, its expression in the brain parenchyma remains unclear. We found that *Cxcl16* increases during AD aging through scRNA. *Cxcr6* is also highly expressed in T cells. Targeting *Cxcr6* to block T cell entry into the brain is a potential therapeutic approach.

Our results corroborate these findings. We functionally annotated the core genes for their biological processes (Supplementary data 8). GO analysis (Fig. 3E) showed that the top 20 biological processes were immune-related. Negative regulation of the lymphocyte apoptotic process, positive regulation of the response to cytokine stimulus, and response to interferon-gamma were the top 3 processes. This suggests that microglia play an important role in immune regulation during aging and AD progression. Furthermore, we analyzed the different functions of aging in WT and 5× FAD mice (Fig. S4).

### 3.4 APP and CSF signals driven 5× FAD mice to deviate from aging track to AD occurrence

Studying the specific crosstalk between signaling pathways in cells may better resolve the communication between signals (Fig. 2B). Therefore, we compared the changes in signaling crosstalk in microglia during aging and AD progression.

In WT mice, from 2 to 12 and then to 24 months, the interaction signals of microglial CCL and Junctional adhesion molecule (JAM) were first enhanced and then weakened. Compared to 2-month-old mice, the outgoing signal of MHC-1 was enhanced in WT mice at 12- and 24-month-old. The outgoing prosaposin (PSAP) signal was enhanced only in WT mice at 24-month-old. The incoming PTN signal decreased with age in the WT mice (Fig. 2B). Abnormally active CCL, JAM, and MHC-1, etc. involved in immune signaling suggests that immune regulation in the body plays a major role in normal aging. Additionally, the weakened signal of PTN, a developmentally regulated trophic factor, and the enhanced signal of PSAP, a trophic factor and activator protein for sphingolipid hydrolase in lysosomes, may be involved in this immune regulation during normal aging.

In 5× FAD mice, the CCL interaction signal decreased with age (Fig. 2B). From 2 to 12 and 24 months, the interaction signal flow of CSF, MHC-1, insulin-like growth factor (IGF), Thy-1 membrane glycoprotein (THY1), and sphingosine-1-phosphate phosphatase 1 (SPP1) was first enhanced and then weakened in 5× FAD mice. The interaction of APP was equally strong at 12- and 24-month-old and weakest at 2-month-old in 5× FAD mice. The interaction of JAM was equal at 2- and 24-month-old and was strongest at 12-month-old in 5× FAD mice. The incoming midkine (MK) signal was strongest at 2-month-old and equally weakened at 12- and 24-month-old in 5× FAD mice (Fig. 2B). This indicated that there was a difference in the signal crosstalk in microglia with aging between normal WT and 5× FAD mice.

Compared with age-matched WT mice, the interaction signal of CCL was weakened in 5× FAD mice (Fig. 2B). The incoming signal of the CSF was weakened at 2-month-old while enhanced in 5× FAD mice at 12- and 24-month-old compared to age-matched WT mice. Compared with age-matched WT mice, the outcome signal of MHC-1 and the incoming signal of APP were enhanced in 5× FAD mice at 12- and 24-month-old. Additionally, PTN, PSAP, IGF, macrophage migration inhibitory factor, SPP1, MK, and progranulin were involved in the abnormal signal crosstalk of microglia in the hippocampus of 5× FAD mice (Fig. 2B). This suggests that these signaling pathways are important in the pathogenesis of AD.

We found that the interaction of the CCL, MHC-1, JAM, PTN, and PSAP signaling pathways was involved in the normal aging of WT mice. The crosstalk between CCL, MHC-1, JAM, CSF, IGF, SPP1, APP, MK, and THY1 plays a key role in the aging of AD model 5× FAD mice. Compared to the three age-matched WT mice, the signaling interactions of CCL, MHC-1, CSF, and APP played a core role in the progression of AD. This indicated that the signaling crosstalk between CCL and MHC-1 was the bridge of communication between microglia and the other 10 cell types. APP and CSF signals drove 5× FAD mice to deviate from the aging track to the occurrence of AD (Supplementary data 6).

### 3.5 Microglia involving in the progression of aging and AD can be divided into 10 unique feature types according to function

**Fig. 4:**
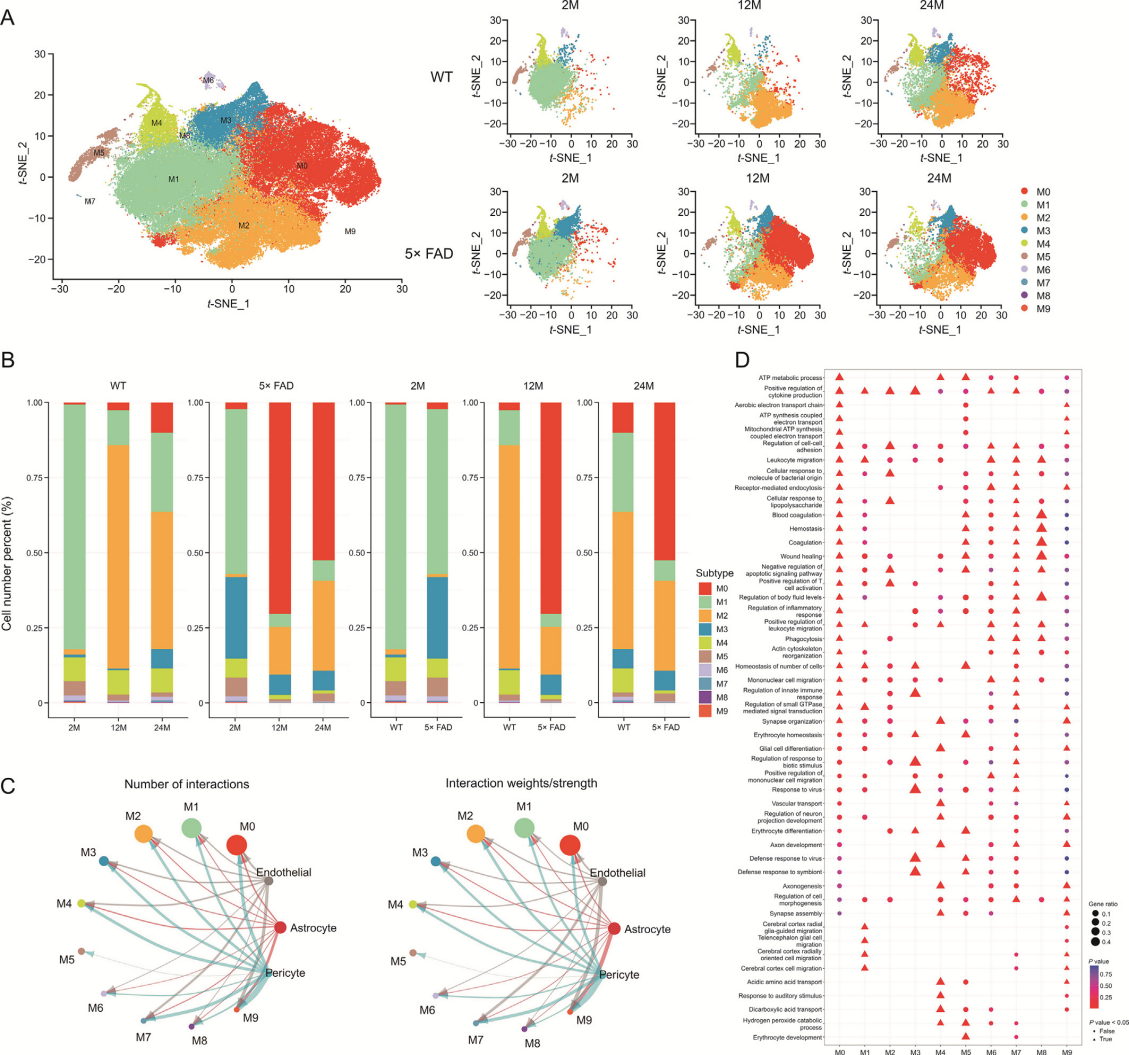
Number and function of microglia subcluster. (A) *t*-Stochastic neighbor embedding (*t*-SNE) dimensional reduction and cell-type interpretation of 90869 microglia in the hippocampus of 2-, 12-, and 24-month-old wild type (WT) and 5× familiar Alzheimer's disease (5× FAD) mice (*n* = 3). (B) The stacked bar chart shows the relative proportion of each microglial subcluster for each biological group (*n* = 3). (C) The blood-brain barrier (BBB) emits signaling that affects microglial subtypes. (D) Gene Ontology (GO) analysis annotated the biological process of microglia subcluster. ATP: adenosine triphosphate.

To further clarify the specific subsets of microglia that play a role in aging and AD progression, we divided microglia into 10 subtypes at 0.2 resolution (Fig. 4A). Combining the *t*-SNE displayed by group (Fig. 4A) and stacked bar chart (Fig. 4B), the M0 subtype gradually increased in WT mice with age. In 5× FAD mice, from 2 to 12 and then to 24 months, the M0 subtype gradually first increased and then decreased and was significantly more than one at the age of 2 months. Additionally, compared with age-matched WT mice, the M0 subtype was markedly increased in 5× FAD mice. The M0 subtype was the most pronounced in 12-month-old 5× FAD mice.

From 2 to 12 and 24 months, the M1 subtype first decreased, then increased, and was significantly less than one at the age of 2 months in WT and 5× FAD mice. The M1 subtype gradually decreased in 5× FAD mice compared to age-matched WT mice. The M1 subtype was most pronounced in 2-month-old WT mice (Fig. 4B).

In WT mice from 2- to 12- and 24-month of age, the M2 subtype first increased and then decreased and was significantly more than 2-month of age. The M2 subtype gradually increased in 5× FAD mice with age. Compared with age-matched WT mice, the M2 subtype gradually decreased in 5× FAD mice. The M2 subtype was most pronounced in the 12-month-old WT mice (Fig. 4B).

In WT mice, from 2 to 12 and 24 months, the M3 subtype first decreased, then increased, and was significantly more at 24-month-old than 2-month-old. The M3 subtype gradually decreased in 5× FAD mice with aging. Compared to age-matched WT mice, the M3 subtype increased in 5× FAD mice until it was approximately equal at 24 months. The M3 subtype was most pronounced in 2-month-old 5× FAD mice (Fig. 4B).

In WT mice, from 2 to 12 and 24 months, the M4 subtype first decreased and then increased and was equal in 24- and 2-month-old mice. The M4 subtype gradually decreased in 5× FAD mice with age. Compared with age-matched WT mice, the M4 subtype gradually decreased in 5× FAD mice. The M4 subtype was the most pronounced in 12-month-old WT mice (Fig. 4B).

There were fewer than 1000 cells in subtypes M5, M6, M7, M8, and M9. They showed different changes with age in WT and 5× FAD mice (Fig. 4B).

To study the effect of BBB injury on microglial subtypes, we analyzed the signaling flow of BBB-associated cells into microglial subtypes. The three most affected microglial subtypes were M9, M7, and M0. In M9 subtype precursor state cells, communication was significant at 2-month of age and virtually absent at 12- and 24-month. The M0 subtype, a typical pro-inflammatory microglial subtype, responded most strongly to BBB damage and central nervous system (CNS) inflammation (Fig. 4C). The M7 subtype functionally resembled the M0 subtype (Table S1 and Fig. 4D).

These results suggest a transformation of the microglial state. The core genes of each subtype were identified (Supplementary data 9), and the top 10 genes are presented as a heatmap (Fig. S5). This may provide a reference for identifying different microglial states. We used GO analysis to explore the function of microglial subtypes (Fig. 4D), details are shown in Table S1 and Supplementary data 10. These results indicate that microglia play an immunomodulatory role in the progression of aging and that AD can be divided into 10 functional subtypes. They were subtype M0 (typical pro-inflammatory microglia), M1 (glia-guided migration), M2 (inflammatory response induced by a bacterial infection), M3 (viral defense and immune regulation), M4 (interacting with neurons and direct microglial differentiation early in CNS), M5 (BBB injury defense), M6 (phagocytosis-related microglial subtypes), M7 (innate immunity similar to the M0 subtype), M8 (blood coagulation and hemostasis similar to the M5 subtype), and M9 (a precursor type of microglia) (Table S1).

### 3.6 The interaction strength among microglial subtypes became weak with aging, and it was more active immune cell communication in AD

**Fig. 5:**
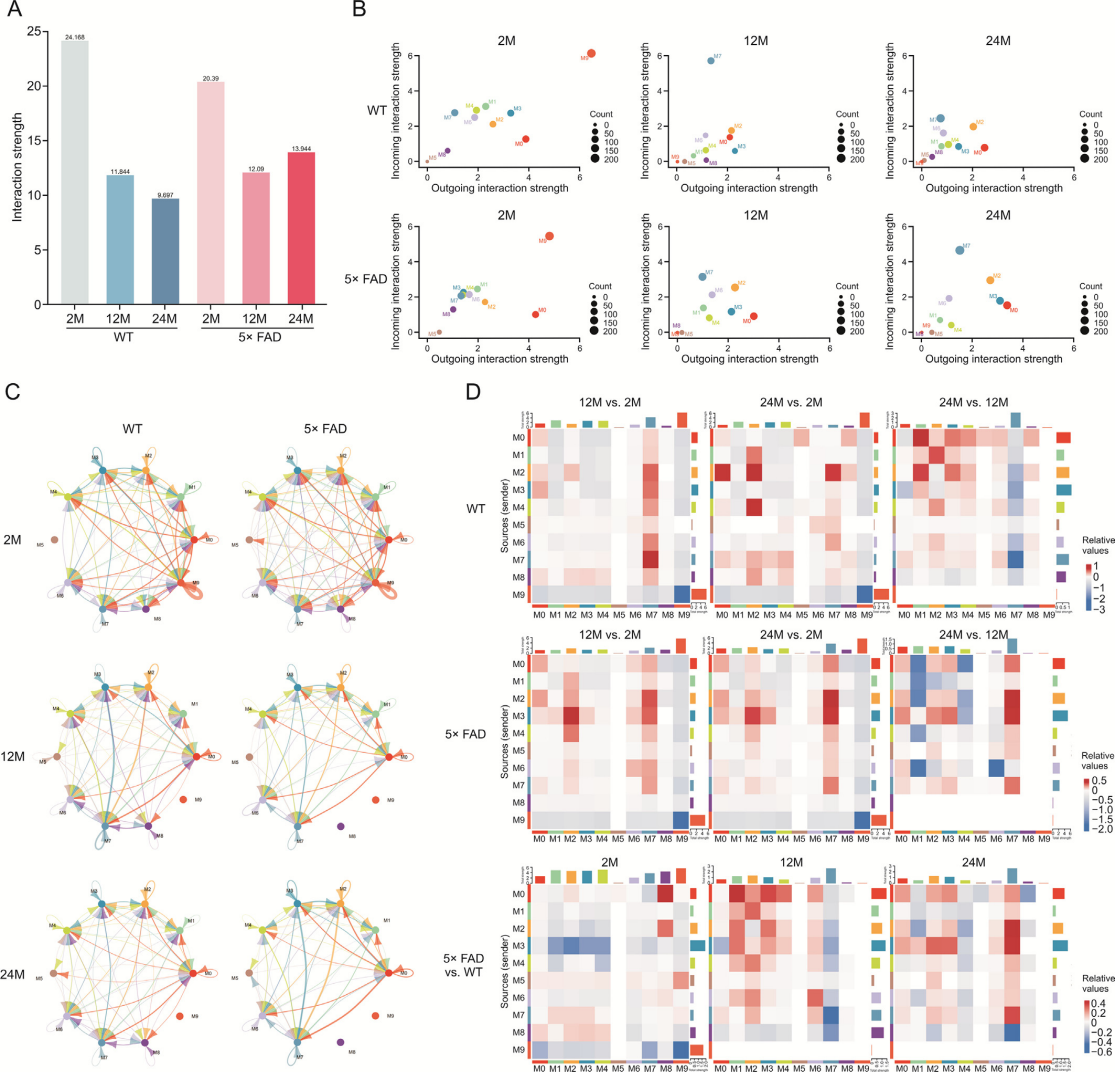
Global differences in cell communication pathways in microglia subcluster were observed among 2-, 12-, and 24-month-old wild type (WT) and 5× familiar Alzheimer's disease (5× FAD) mice. (A) The weighted strength of cell interactions was calculated using Cellchat for microglia in the 2-, 12-, and 24-month-old WT and 5× FAD groups. (B) The bubble diagram shows the outgoing and incoming interaction strength comparison of 10 microglial subclusters in the 2-, 12-, and 24-month-old WT and 5× FAD groups. (C) Circle diagram shows the interaction network of microglia subcluster interaction strength. (D) Heatmaps demonstrate differences in the strength of microglia sub-cluster crosstalk between the groups. The ordinates represent the source subcluster, and the abscissa represents the target subcluster. Red represents increased interaction strength and blue represents decreased interaction strength.

Along with aging and the AD process, microglial subtypes also change. Communication between microglial subtypes may help better analyze the function of microglia (Supplementary data 11). We observed the total number of interactions (Fig. S6A) and the strength (Fig. 5A) of the microglial subtype. In WT mice, the strength of this interaction gradually weakens with age. In 5× FAD mice, the interaction strengths at 12 and 24 months were lower than those at 2 months. However, compared with that at 12 months, the interaction strength at 24 months was slightly elevated. At 2-month of age, the interaction strength of the WT was higher than that of 5× FAD, but at 12- and 24-month of age, the interaction strength of the WT was lower than that of 5× FAD (Fig. 5A). This suggests the activation of immune cell communication during AD.

Based on the overall interaction strength of each subtype (Fig. 5B), M0, M2, M3, M6, and M7 had strong interaction strengths in each group and were the active and functionally predominant subtypes. The M9 subtype had a strong communication function at the age of 2 months and disappeared with aging in WT and 5× FAD mice. The incoming interaction strength of the M7 subtype in WT mice at 12-month of age was significantly higher than those at 2- and 24-month of age. This was positively correlated with aging in 5× FAD mice. The M0 subtype had the highest outgoing interaction strength at 2 months of age, and the outgoing interaction strength was relatively weak at 12- and 24-month of age in both mouse strains. However, compared with age-matched WT mice, the M0 outgoing signal was stronger in 5× FAD mice. The interaction strength of the M2 subtype did not change significantly with age in the WT mice, but the cross-signal intensity increased slightly with age in the 5× FAD mice. The interaction strength of the M5 subtype was low. Similarly, the interaction strength of the M8 subtype in WT mice was always low and significantly decreased at 12- and 24-month of age in 5× FAD mice. For the M3 subtype, the interaction strength in WT mice decreased with age. In 5× FAD mice, the outgoing interaction strength increased with age. Whether in WT or 5× FAD mice, the incoming and outgoing interaction strength of the M1 and M4 subtypes at the age of 2 months was higher than that in the elderly. There was almost no change in the strength of the interaction with the M6 subtype (Fig. 5B).

The circle diagram (Fig. 5C) shows the interaction network of the microglial sub-cluster interaction strength. We found that the strength of the interaction between the subtypes weakened with age. The number of interactions decreased gradually (Fig. S6B). We compared the differences in the interaction strength of each subtype with aging in WT and 5× FAD mice (Fig. 5D). In WT or 5× FAD mice, compared to 2-month-age, the overall crosstalk intensity decreased at 12-month-old and 24-month-old (Fig. 5A), but the incoming M7 was enhanced (Fig. 5D).

### 3.7 The signaling pathways of CCL and CSF were the fundamental bridge of communication among microglia subtypes

**Fig. 6:**
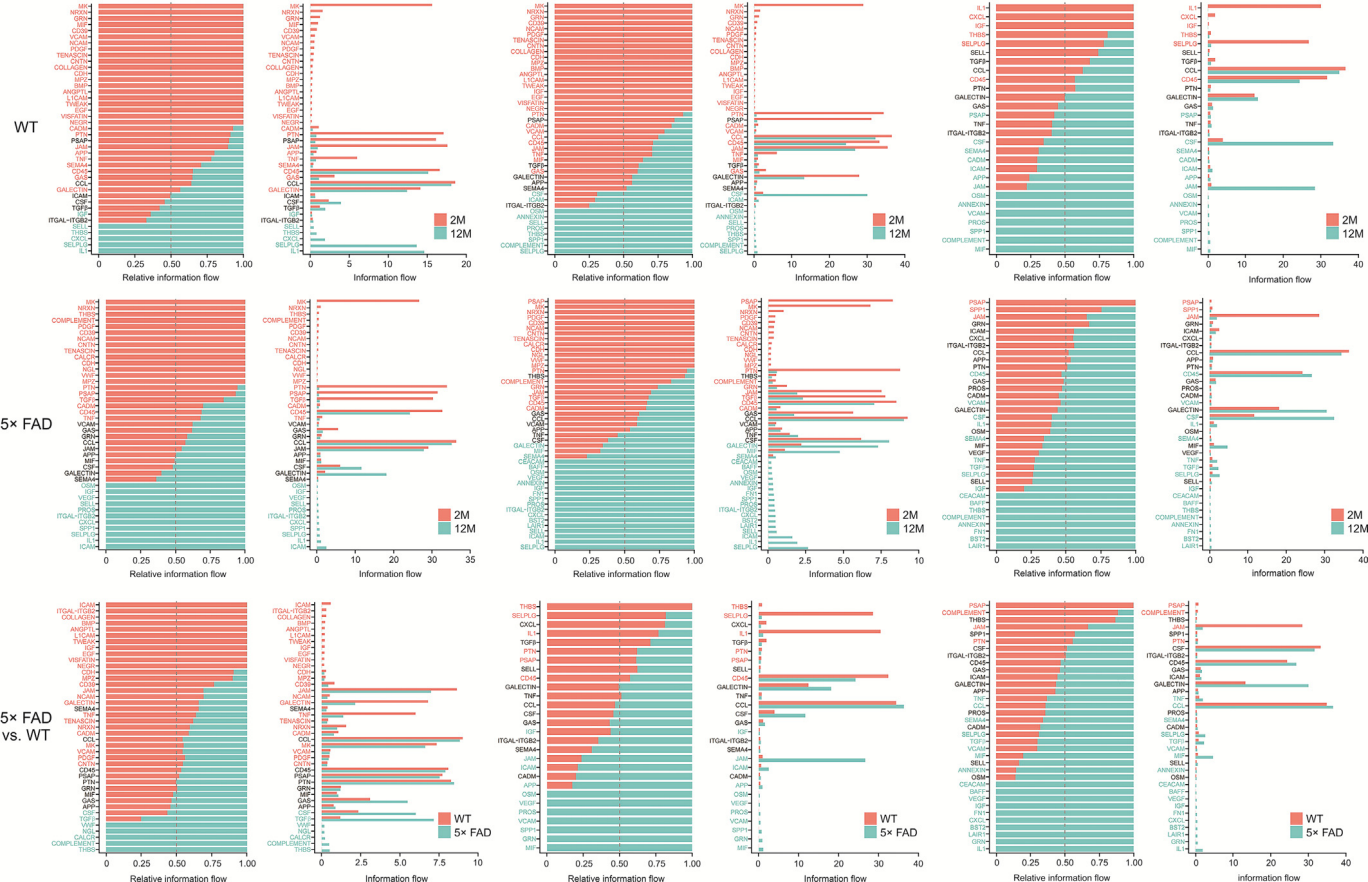
Signaling changes comparison between two groups based on the information flow. WT: wild type; 5× FAD: 5× familiar Alzheimer's.

**Fig. 7:**
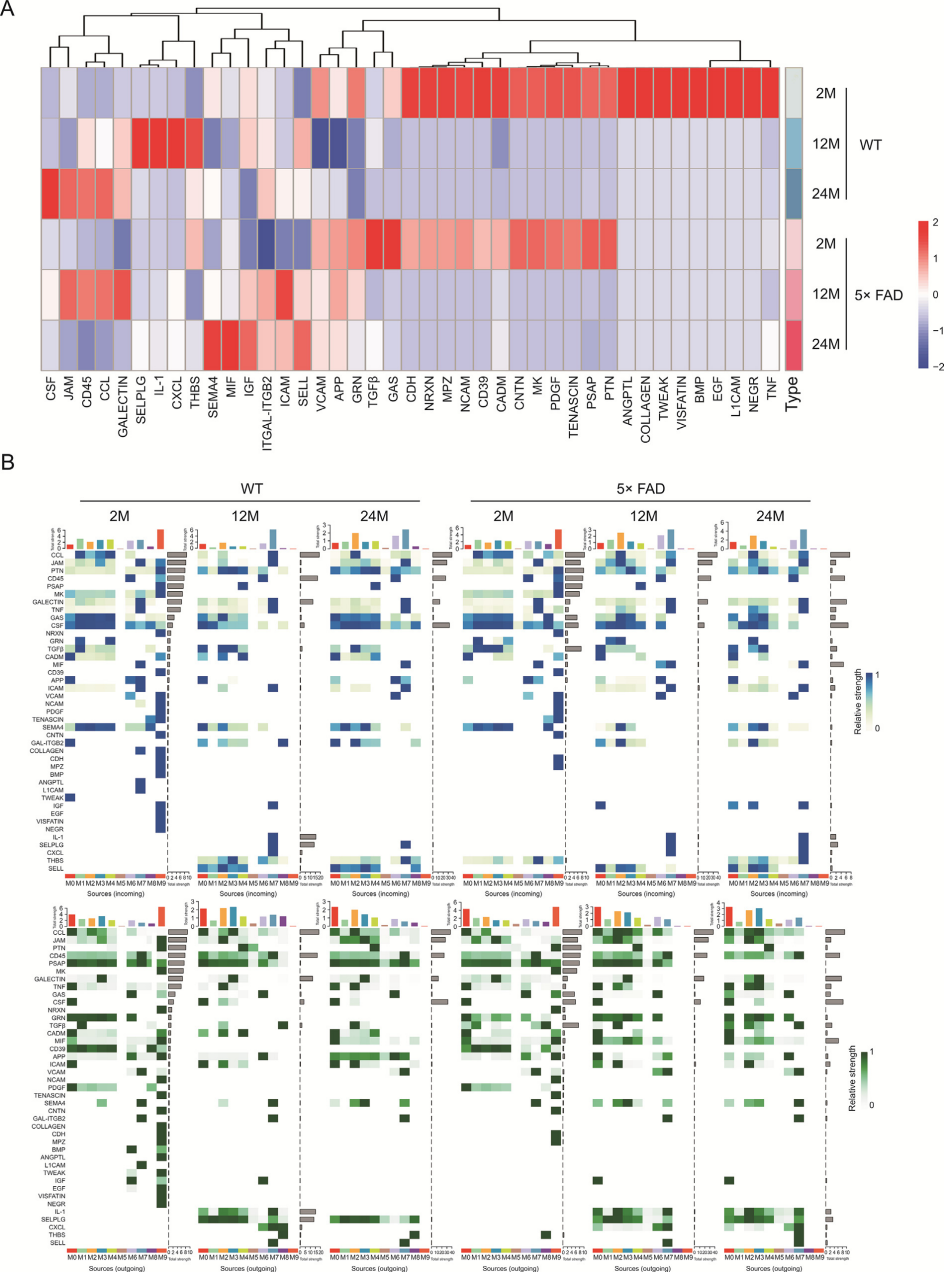
Signaling changes of microglia subcluster in wild type (WT) and 5× familiar Alzheimer's disease (5× FAD) mice during different ages. (A) Heatmaps show the information flow variation trends in each group on the whole. (B) Identification of the signal contribution of outgoing and incoming signaling pathways in the microglia subcluster within 2-, 12-, and 24-month-old WT and 5× FAD groups is depicted by a complex heatmap. Incoming signaling is represented in blue and outgoing signaling is in green. TNF: tumor necrosis factor; NEGR: neuronal growth regulator; L1CAM: neural cell adhesion molecule L1; EGF: epidermal growth factor; BMP: bone morphogenetic protein; TWEAK: tumor necrosis factor receptor superfamily; PTN: pleiotrophin; PSAP: prosaposin; PDGF: platelet-derived growth factor; MK: midkine; CNTN: contactin; CADM: cell adhesion molecule; NCAM: neural cell adhesion molecule; MPZ: myelin protein P0; NRXN: neurexin; CDH: cadherin; GAS: growth arrest-specific protein; TGFβ: transforming growth factor β; GRN: progranulin; APP: amyloid precursor protein; VCAM: vascular cell adhesion protein; SELL: l-selectin; ICAM: intercellular adhesion molecule; ITGAL: integrin alpha-L; ITGB2: integrin beta-2; IGF: insulin-like growth factor; MIF: macrophage migration inhibitory factor; SEMA4: semaphorin-4; THBS: thrombospondin; CXCL: C−X−C motif chemokine ligand; IL-1: interleukin-1; SELPLG: P-selectin glycoprotein ligand; CCL: C−C motif chemokine ligand; JAM: junctional adhesion molecule; CSF: colony-stimulating factor.

**Fig. 8:**
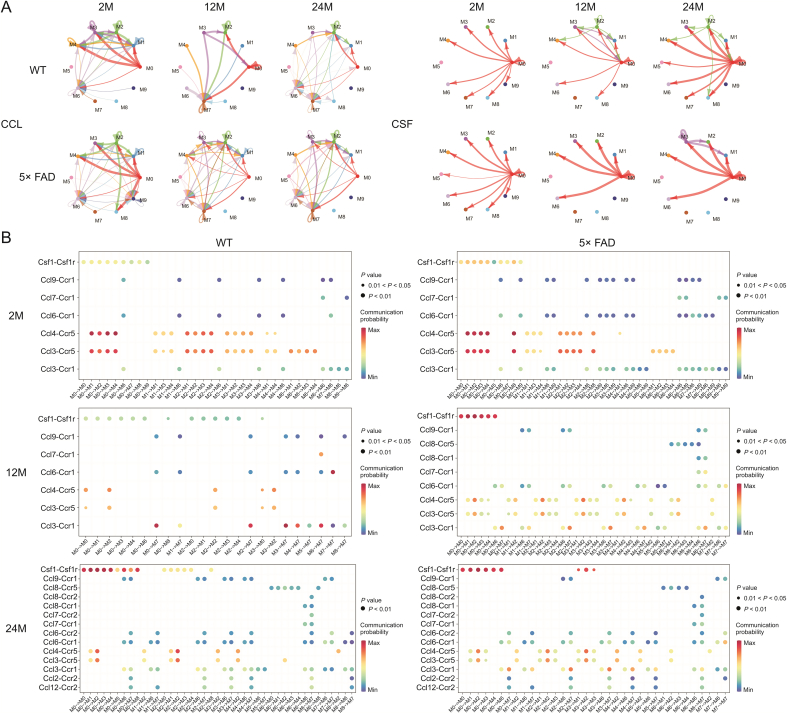
Interaction between C−C motif chemokine ligand (CCL) and Colony-stimulating factor (CSF) signal subtypes. (A) Crosstalk mode between subtypes of CCL and CSF signal. (B) Ligand-receptors contribute in CCL and CSF signals to crosstalk. WT: wild type; 5× FAD: 5× familiar Alzheimer's.

To further explore the specific information flow in aging and AD, we compared the strength of information flow in all 10 microglial subtypes (Fig. 6). The CCL signaling pathway was strongest at 24-month of age in WT mice and peaked at 12-month of age in 5× FAD mice (Fig. 7A). Interestingly phenomenon was discovered and, regardless of strain or age, the CCL pathway exhibited strong interaction strength (Fig. 6, Fig. 7). This suggests that CCL may play a mainstay role in the self-communication of microglia. Regarding incoming signaling (Fig. 7B), CCL was emitted by different microglial subtypes at different stages. In WT mice, the strongest CCL signaling cell types were M1, M7, and M2 at 2-, 12-, and 24-month of age. In 5× FAD mice, the strongest CCL signaling cell types were M1, M2, and M2 at 2-, 12-, and 24-month of age. Regarding outgoing CCL signaling (Fig. 7B), the M0 subtype was strongest at 2 months of age in WT and 5× FAD mice and then gradually weakened. In WT mice at 12-month-old, M3 showed a strong CCL signal, and at 24-month-old, M2 showed the strongest signal. In 5× FAD mice, M2 and M3 macrophages play a strong role. In addition, we visualized crosstalk between each subtype in the CCL signaling pathway (Fig. 8A). We explored the specific receptor ligands in the CCL signaling pathway and found that CCL2, CCL12, and C−C motif chemokine receptor 2 (CCR2) appeared only at 24 months. CCL8 appeared at 24 months in WT mice and at 12 months in 5× FAD mice. CCL8 was predominantly produced by the M6 subtype (Fig. 8B). Based on the function of the M6 subtype (Table S1 and Fig. 4D), it was speculated that it might be a signal for the recruitment of other subtypes after the M6 subtype exerts its phagocytic effect. This pathway may also serve as a chemotactic pathway for microglial senescence.

In WT and 5× FAD mice, the strength of the CSF signaling pathway increased with age. Microglia depend on CSF1 for their differentiation and survival, and CSF 1 receptor (CSF1R) inhibition alters macrophage polarization [32]. We also found that the number of microglia gradually increased (Figs. 1A–C). The gene expression level of *Csf1* increased with age and was higher in 5× FAD mice than in age-matched WT mice (Fig. 3C). Among the microglial subtypes, the M0 subtype increased the most significantly with age (Fig. 4B). The CSF signaling pathway was mainly mediated by M0 (Fig. 8A). We studied the receptor-ligand pairs of each microglial subtype. Then it was found that the CSF1-CSF1R expression of the M0 subtype was the highest (Fig. 8B).

Additionally, the transforming growth factor beta (TGFβ) signal was found to be strongest in 5× FAD mice at 2-month of age (Fig. 7A), mainly outgoing by M8 (Fig. 7B). Receptor-type tyrosine-protein phosphatase C (CD45) is a signaling pathway that increases with age in WT mice, whereas in 5× FAD mice, the signal exchange peaks at 12-month of age.

## 4 Discussion

### 4.1 Microglial proliferation and activation induce cognitive impairment

In rodents, microglia account for 5%–12% of all CNS cells and are distributed throughout the parenchyma [33]. We found that microglia made up over 12% of the total hippocampal cells, about 45%–55% in WT mice and 45%–78% in 5× FAD mice. Maintenance of the brain microglial population is independent of circulating monocytes generated in the bone marrow and depends primarily on the self-renewal of microglia. A study on microglial depletion showed that microglia form clusters of highly proliferative cells with the help of interleukin (IL)-1 receptor signaling and migrate after reaching a steady state [34]. Our scRNA-seq results showed that in WT mice, the number of microglia increased significantly at 24 months, but the fraction of microglia in the total hippocampal cells remained stable. In the hippocampus of 5× FAD mice, the number and proportion of microglia increased abnormally at 12 months, and at 24 months, the number of microglia tended to level off compared with the 12-month of age. The accumulation of Aβ also showed a similar trend. This may be one of the reasons for the induction of microglial activation and proliferation. In addition, from 2 to 24 months, the PTN growth signal received by microglia in WT mice tended to decrease, suggesting that excessive growth of microglia was continuously suppressed. In 5× FAD mice, the growth and development signals of PTN, CSF, and JAM were significantly enhanced at 12 months compared to those at 2 months. The uncontrolled proliferation of microglia is clearly not spontaneous but arises from environmental stimuli.

Microglia play an important role in synapse pruning, stimulation of learning-related synapse formation in the healthy adult brain, and maintenance of neuronal connectivity and synaptic homeostasis for learning and memory [35,36]. In addition, microglia play a major role in AD, and their overactivation substantially increases the production of cytokines and reactive oxygen species [9,37].

Microglial activation is a key neuropathological signature of AD. The glial response occurs around early Aβ plaques and tau tangles, and reactive glial cells may exacerbate ongoing neurodegeneration [38,39]. Neuroinflammation drives neurodegenerative diseases such as AD [40,41]. Furthermore, abnormal activation of microglia-mediated T-cell infiltration drives neurodegeneration in tauopathy [42].

Microglia absorb and break down seed-bearing tau and induce activation [43]. Later, tau may be repackaged into exosomes or indirectly involved in the enhancement of tau phosphorylation through pro-inflammatory cytokine signaling [44]. It has been shown that microglial phenotypic changes precede the formation of significant NFT and are predictors of cognitive impairment in individuals with Braak III−IV [45].

The 24-month-old WT mice showed an increase in the number of microglia and a decrease in signal strength. It has been hypothesized that repeated systemic immune challenges induce chronic neuroinflammation and accelerate microglial aging. Senescent microglia (initially considered highly reactive) may show a decrease in responsiveness to injury signals over time, further worsening Aβ accumulation and NFT forms toxicity [46]. Microglial cells in the aging brain have fewer branches, thereby reducing their surveillance area, which may lead to impaired homeostatic functions in inflammation regulation [47]. In 24-month-old 5× FAD mice, the accumulation of microglial cells did not persist, but tended to stabilize, which may also be related to the reduced reactivity of microglial cells to damage and inflammation after aging.

### 4.2 Neuroinflammation induced by BBB-related cell injury promotes abnormal proliferation of microglia

In this study, GO analysis of 41 common differential genes in the six groups revealed that they were primarily involved in the production of chemokines, cytokines, IL-6, and other molecules, as well as in neuro-inflammatory processes such as lymphocyte apoptosis and adaptive immune regulation. The inflammatory microenvironment of the hippocampus is the key reason for differences in the phenotypic differentiation of glial cells. These inflammatory mediators are produced by resident central immune cells, BBB, and peripheral immune cells [48]. In our study, microglia communicated closely with BBB cells, especially in the 5× FAD mice. Pericytes, endothelial cells, and astrocytes transfer growth factor PTN to microglia. The BBB does not operate independently but as a module of the multicellular neurovascular unit (NVU). A large amount of intercellular communication occurs between the cells of the vascular system, adjacent neurons, and glial cells [49]. Resident microglia use long cellular processes to measure the microenvironment of the vascular basement membrane and respond quickly to damage at or near the NVU [49]. Under pathological conditions, microglia around the blood vessels are activated and migrate to the BBB, inducing neuroinflammation and exacerbating BBB damage. In the brain of ischemic stroke patients, necrotic neurons and glial cells release inflammatory signals or damage-related molecular patterns and activate resting pericytes and microglial cells [49]. Further recruitment of peripheral immune cells and upregulation of matrix metalloproteinases lead to impairment of the BBB [50]. Within 24 h of resumption of blood perfusion, activated microglia also phagocytose endothelial cells, leading to vascular disintegration and expansion of the blood-stasis zone [51]. Considering the close communication between pericytes and microglia, whether the microglial phenotype can be acquired during BBB repair remains unclear. Among all the microglial subtypes, the M9, M0, and M7 subtypes received the strongest signal from the BBB. This suggests that the cell subtypes with anti-inflammatory functions are vulnerable to stimulus activation, thus providing a reference for the activation of microglia specific to the central inflammatory response.

### 4.3 Immunomodulatory imbalance in aging microglia

The 24-month-old WT mice showed an increase in the number of microglia and a decrease in signal strength. It has been hypothesized that repeated systemic immune challenges induce chronic neuroinflammation and accelerate microglial aging. Senescent microglia (initially considered highly reactive) may show a decrease in responsiveness to injury signals over time, further worsening Aβ accumulation and NFT forms toxicity [46]. Microglial cells in the aging brain have fewer branches, thereby reducing their surveillance area, which may lead to impaired homeostatic functions in inflammation regulation [47]. In 24-month-old 5× FAD mice, the accumulation of microglial cells did not persist, but tended to stabilize, which may also be related to the reduced reactivity of microglial cells to damage and inflammation after aging.

### 4.4 MHC-I and CCL were the fundamental bridge for microglia to participate in neuro-immunity in the process of aging and AD

At 12-month of age, 5× FAD mice showed enhanced MHC-1 signaling between hippocampal microglia and T cells compared to WT mice, indicating stronger central immune infiltration in 5× FAD mice. The slow turnover rate and long lifespan of microglia make them perfect host depots for CNS viruses [52]. After viral colonization, microglia release cytokines, chemokines, and neurotoxic mediators that trigger pathological neuroinflammation [53].

The antigen presentation function of MHC molecules is crucial for the activation of CD8^+^ T cells to differentiate into effector cytotoxic T lymphocytes (CTLs). Under the action of chemokines, CTL leaves the lymphoid tissue and accumulates at the site of infection. Microglia mediate T-cell entry into the parenchyma during neuroinflammation [54]. From another perspective, microglia prevent the spread of the virus in the CNS and act as guards for neurons [55]. Aging brains also exhibit T-cell infiltration in the neurogenic niche and impaired neural stem cell proliferation, which may explain the decline in brain function with aging [56]. CCL signal flow has a chemotactic effect on immune cells, such as neutrophils and monocytes. The CCR is expressed on the surface of microglia [57]. T-cells exert chemotactic effects on microglia throughout life. In addition, interferons secreted by effector T cells can enhance the expression of MHC-I/II in microglia and enhance their phagocytic effect [58]. These results indicated their synergy in eradicating pathogenic infections.

### 4.5 APP and CSF signals drive 5× FAD to deviate from aging track to AD occurrence

Compared to WT mice at 12- and 24-month of age, 5× FAD mice showed stronger signaling interactions between microglia and the hippocampal microenvironment, where APP and CSF may play a significant role. APP, a type I transmembrane protein, is a precursor molecule of Aβ production [59]. At 2 months, microglia presented with human leukocyte antigen class II histocompatibility antigen gamma chain **(**CD74) receptors on the surface of T cells. In 5× FAD mice at 12 and 24 months, excessive APP is secreted or expressed by oligodendrocytes, endothelial cells, pericytes, and ependymal cells, interacting with CD74 receptors on the surface of microglial cells. CD74 is a type II transmembrane protein of 216 amino acids that acts as a molecular chaperone in the MHC-II [60]. CD74 interacts with APP and aggregates it into endocytic vacuoles generated by CD74, thereby inhibiting Aβ production by regulating the subcellular localization of APP [61]. Recombinant adeno-associated virus delivers CD74 to the hippocampus of AD mice, reducing the Aβ load in the hippocampus and Aβ accumulation in pyramidal neurons [62]. Although it does not alter the final outcome, the expression of CD74 on the surface of microglia may be a negative feedback mechanism of the brain against Aβ production.

CSF1, a strong proliferation signal for microglia, was significantly enhanced in WT mice at 24-month old and in 5× FAD mice at 12-month of age. Immunohistochemical staining showed that CSF1R was expressed only in the microglia of the postnatal mouse brain [63]. CSF1R was also expressed in the neural stem cells of 5× FAD mice at 12 months, which was induced by microglial cells to further expand the microglial army. CSF1 is essential for the development of microglia and the maintenance of normal brain structures in early life [63]. However, in adulthood, spikes in CSF signals appear to exacerbate the damage to neurological function. Evidence suggests that active CSF1 signaling mediates reactive microglial destinations [63]. Chronic microglial depletion (inhibited by CSF1R) can significantly increase peripheral neural networks and synaptic connections with excitatory cortical neurons [64]. It also reduces the levels of inflammatory mediators in the hippocampus, the recruitment of CCR2 white blood cells, and circulating inflammatory factors [65]. Interestingly, we found that CSF1-CSF1R signal transduction occurred only in glial cells and that it interacted with neural stem cells. Most CSF signals received by microglia originate from pericytes and are mediated by IL-34-CSF1R. There are no reports of pericytes secreting IL-34. During early development, microglial precursors are attracted to proximal brain regions by brain-derived IL-34 [66], suggesting that IL-34 emitted by pericytes may regulate microglial migration and colonization of blood vessels.

### 4.6 IGF revealed the protective effect of microglia cells on CNS

IGF plays a trophic role in development and tissue damage, and microglial cells are an important source of IGF1 [67]. In 5× FAD mice at 12- and 24-month of age, IGF1 was secreted by microglia into oligodendrocytes, endothelial cells, and pericytes. Pericytes interact with the IGF1 receptor on the surface of microglia via IGF2. IGF1 secreted by astrocytes induces oligodendrocyte growth and myelination. Vitro experiments showed that IGF2 could inhibit oligodendrocyte death induced by tumor necrosis factor-alpha [68]. Although the exact mechanism remains unknown, current evidence suggests that IGF signaling is mediated by microglia, which exert protective effects on the BBB.

### 4.7 Signal of CCL and CSF as the fundamental bridge affecting the crosstalk from microglia subtype

CSF signaling regulates microglial proliferation during chronic neurodegeneration [25]. At the age of 12 months in 5× FAD mice and 24 months in WT mice, the CSF signal peaks during communication between microglial subtypes. This is considered as a period of high-speed proliferation of microglia, and our statistics on the number and proportion of microglial subtypes also proved this point. We found that the CSF is mainly mediated by the M0 subtype, the dominant subtype of abnormal microglial proliferation and neuroinflammation in AD. *Cd74, Igf1*, and the high-genetic-risk gene *Apoe* of AD are all marker genes specifically expressed in the M0 subtype (Supplementary data 9). In addition, the marker genes of *Cd11c* microglial cells are highly expressed, including *Spp1*, *Cst7*, *Csf1*, and *Lpl*, which are microglial cells related to neurodegenerative diseases and stroke injuries [[69], [70], [71]]. *Cd11c* has been considered as a marker of dendritic cells and is a member of the leukocyte adhesion molecule β2 integrin, which can promote leukocyte recruitment, phagocytosis, and immune synapse formation [72]. *Cd11c* is also associated with the presentation of T cells [73]. After swallow apoptotic cells, the expression of *Cd11c* of microglia was increased [74]. The number of *Cd11c*^+^ microglia reached its peak in early postnatal life (P3−P5). Then it drops to the edge level in early adulthood and proliferate again during normal aging [70]. *Cd11c* microglia also have anti-inflammatory activities, such as promoting recovery from neuropathic pain by expressing *Igf1* [75]. Furthermore, *Cd11c* microglia is accompanied by the expression of the *Apoe* gene [69]. *Apoe* may promote plaque seeding in the initial stage of plaque formation, aggravate plaque deposition, and act as an opsonin to enhance plaque phagocytosis by microglia [76]. The M0 subtype has the characteristics of *Cd11c* microglia, which appear after CNS injury or during neuroinflammation and regulate immunity and phagocytosis.

CCL signaling is consistently robust across subtypes of global perturbations. Its signaling interaction is mainly concentrated in the M0−M4 subtype. This indicates a close interaction between the two. In this study, seven ligands were identified: CCL3, CCL4, CCL6, CCL7, CCL9, CCL8, and CCL12. CCL8 appeared at 24 months in WT mice and 12 and 24 months in 5× FAD. They are also emitted by the M6 subtype. M6 isoforms are associated with CD45 signaling, which may be related to elevated phagocytic activity [77]. At the same time, we noticed that the M6 subtype sent growth arrest-specific protein 6 (GAS6) signals to other subtypes at 2 months in WT mice, and at 2 and 24 months in 5× FAD mice. At the age of 2 months, only a small number of Aβ plaques were observed in 5× FAD mice. Therefore, it is believed that M6 plays different roles in sending GAS6 signals at 2- or 24-month of age. At 2-month of age, this may be related to the GAS6-tumor-associated macrophage (TAM) promoting myelination and glial cell development in the CNS [78]. At 24-month of age, the GAS6-TAM system may be an important mediator of microglial recognition and phagocytosis of Aβ plaques, although TAM-driven microglial phagocytosis cannot form plaques [79]. At the same time, GAS6 is also a response factor of microglial cells to brain injury stimuli [80]. This suggests that the M6 subtype may be an effector of microglial phagocytosis. CCR1, CCR2, and CCR5 are involved in the CCL signaling pathway.

Among them, CCR5 had the highest signal strength (the highest at the age of 2 months), and CCR2 (ligands CCL2 and CCL12) appeared in WT and 5× FAD mice at the age of 24 months. CCR5 is involved in microglial chemotaxis in an in vivo model, CCR5 antagonists significantly inhibited the migration of macrophages to brain tissue after lipopolysaccharide treatment [81]. Interestingly, systemic inflammation induces the CCR5-dependent migration of brain microglia to the cerebrovascular system to maintain the BBB [82]. Thus, CCR5 is a therapeutic target for recovery after stroke and traumatic brain injury [83]. In the specific receptor-ligand communication of CCR5, the M2 subtype is prominent. The M2 subtype is associated with bacterium-induced immunity. Monocytes adhere to the endothelial cells of the cerebral vessels and carry bacteria from the periphery into the brain parenchyma via cell spreading [84]. Endothelial are the main constituents of the BBB. In the overall crosstalk between microglia and other cells, we found that BBB damage may be the main pathway leading to abnormal proliferation of microglia and an inflammatory response. When the BBB is disrupted and endothelial cells carry bacteria into the brain parenchyma, the M2 subtype is primarily recruited to eliminate the bacterial inflammatory responses. CCR2 may mediate peripheral monocyte and macrophage infiltration of the CNS. Initially, CCR2 was limited to circulating immune cells (monocytes/macrophages) and not expressed by microglia [85]. In clinical samples, the CCL2 protein is locally produced within a few hours of acute traumatic brain injury and continues to rise in the cerebrospinal fluid for up to nine days [86], which may signal the peripheral recruitment of CCR2 immune cells. Mice lacking CCR2 showed reduced macrophage infiltration into the hippocampus, enhanced neuronal survival, and improved cognitive outcomes [87]. CCR2 may also be involved in microglial-macrophage interactions. Microglia recruit monocytes to damaged brain regions through CCL2 expression and then differentiate into monocytes/macrophages via direct interferon-1 responses in a subset of microglia [88]. In our study, CCR2 was expressed by M7-type microglia. Additional experimental studies are required to determine whether M7-type microglia can differentiate from peripheral monocytes/macrophages. The M9 subtype acts as a microglial precursor and disappears during aging. However, M8 only functioned in aging WT mice. TGFβ signal (TGFβ1) output by M8 subtype may be a restraint signal between subtypes, which was heavily expressed in 12-month WT and 2-month 5× FAD, but was substituted by M2 and M7 subtypes at 24-month WT and 12-month 5× FAD, and the intensity was weakened. The disappearance of the M8 subtype TGFβ1 signal and the total TGFβ1 signal trough were consistent with the uncontrolled proliferation time of microglia. TGFβ1 may be one of the most effective endogenous factors regulating microglia function, playing a key role in promoting microglia maturation, inducing microglia-specific gene expression, and preventing excessive activation of microglia under physiological conditions [89]. Mouse models with reduced levels of active TGFβ1 show inflammation and even tumors [90]. TGFβ1^−/−^ mice showed strong microglial proliferation and cortical neuronal degeneration [91], and loss of microglial maturation markers *P2ry12*, *Sall1*, and *Fcrls* [92]. Tamoxifen-induced loss of TGFβR2 in adult *Sal1* Cre ERT2 [93] and *Cx3cr1* Cre ERT2 [94] mice resulted in the loss of microglial cell branching and upregulation of inflammatory markers. In WT mice, microglial TGFβ signaling decreased with aging [89]. With the progression of AD, the TGFβ signal recovered in 5× FAD mice at 24 months, which may be the potential factor that the microglia-related neuroinflammation in advanced AD mice did not progress.

## 5 Conclusion

This study found an increased percentage of microglia in aging and AD mice. BBB injury might contribute to this increase. Furthermore, immune core regulation genes were found in aging and disease progression. The APP and CSF signals drive 5× FAD mice to deviate from the aging track to AD occurrence through intercellular communication in the hippocampus. Ten microglial subtypes and fundamental bridges of communication signal (CCL and CSF) were identified. This provides a reference for exploring the pathogenesis of AD. However, these studies still require substantial evidence such as the expression of core communication receptors and ligands.

## CRediT author statement

**He Li:** Data curation, Formal analysis, Methodology, Validation, Visualization, Writing - Original draft preparation; **Tianyuan Ye:** Writing - Reviewing and Editing, Funding acquisition, Project administration; **Xingyang Liu:** Project administration, Resources; **Rui Guo:** Investigation, Resources; **Xiuzhao Yang:** Investigation, Software, Data curation; **Yangyi Li:** Investigation, Software, Validation; **Dongmei Qi:** Funding acquisition; **Yihua Wei** and **Yifan Zhu:** Investigation, Supervision; **Lei Wen:** Writing - Reviewing and Editing, Supervision, Resources; **Xiaorui Cheng:** Conceptualization, Investigation, Data curation, Resources, Writing - Reviewing and Editing, Supervision, Funding acquisition.

## Declaration of competing interest

The authors declare that there are no conflicts of interest.

## References

[1] (2022). 2022 Alzheimer’s disease facts and figures. Alzheimers Dement..

[2] Hou Y., Dan X., Babbar M. (2019). Ageing as a risk factor for neurodegenerative disease. Nat. Rev. Neurol..

[3] Glenner G.G., Wong C.W. (1984). Alzheimer’s disease: Initial report of the purification and characterization of a novel cerebrovascular amyloid protein. Biochem. Biophys. Res. Commun..

[4] Nukina N., Ihara Y. (1986). One of the antigenic determinants of paired helical filaments is related to tau protein. J. Biochem..

[5] Corrada M.M., Berlau D.J., Kawas C.H. (2012). A population-based clinicopathological study in the oldest-old: The 90+ study. Curr. Alzheimer Res..

[6] Robinson J.L., Corrada M.M., Kovacs G.G. (2018). Non-Alzheimer’s contributions to dementia and cognitive resilience in The 90+ Study. Acta Neuropathol..

[7] Perez-Nievas B.G., Stein T.D., Tai H.C. (2013). Dissecting phenotypic traits linked to human resilience to Alzheimer’s pathology. Brain.

[8] Barroeta-Espar I., Weinstock L.D., Perez-Nievas B.G. (2019). Distinct cytokine profiles in human brains resilient to Alzheimer’s pathology. Neurobiol. Dis..

[9] Leng F., Edison P. (2021). Neuroinflammation and microglial activation in Alzheimer disease: Where do we go from here?. Nat. Rev. Neurol..

[10] Ransohoff R.M. (2016). A polarizing question: Do M1 and M2 microglia exist?. Nat. Neurosci..

[11] Rangaraju S., Dammer E.B., Raza S.A. (2018). Identification and therapeutic modulation of a pro-inflammatory subset of disease-associated-microglia in Alzheimer’s disease. Mol. Neurodegener..

[12] Plescher M., Seifert G., Hansen J.N. (2018). Plaque-dependent morphological and electrophysiological heterogeneity of microglia in an Alzheimer’s disease mouse model. Glia.

[13] Li H., Wei M., Ye T. (2022). Identification of the molecular subgroups in Alzheimer’s disease by transcriptomic data. Front. Neurol..

[14] Satija R., Farrell J.A., Gennert D. (2015). Spatial reconstruction of single-cell gene expression data. Nat. Biotechnol..

[15] Zhang Q., He Y., Luo N. (2019). Landscape and dynamics of single immune cells in hepatocellular carcinoma. Cell.

[16] Levine J.H., Simonds E.F., Bendall S.C. (2015). Data-driven phenotypic dissection of AML reveals progenitor-like cells that correlate with prognosis. Cell.

[17] Kobak D., Berens P. (2019). The art of using *t*-SNE for single-cell transcriptomics. Nat. Commun..

[18] Ntranos V., Yi L., Melsted P. (2019). A discriminative learning approach to differential expression analysis for single-cell RNA-seq. Nat. Methods.

[19] Jin S., Guerrero-Juarez C.F., Zhang L. (2021). Inference and analysis of cell-cell communication using CellChat. Nat. Commun..

[20] Oakley H., Cole S.L., Logan S. (2006). Intraneuronal β-amyloid aggregates, neurodegeneration, and neuron loss in transgenic mice with five familial Alzheimer’s disease mutations: Potential factors in amyloid plaque formation. J. Neurosci..

[21] Giladi A., Cohen M., Medaglia C. (2020). Dissecting cellular crosstalk by sequencing physically interacting cells. Nat. Biotechnol..

[22] Vento-Tormo R., Efremova M., Botting R.A. (2018). Single-cell reconstruction of the early maternal-fetal interface in humans. Nature.

[23] Sweeney M.D., Zhao Z., Montagne A. (2019). Blood-brain barrier: From physiology to disease and back. Physiol. Rev..

[24] Sweeney M.D., Sagare A.P., Zlokovic B.V. (2018). Blood-brain barrier breakdown in Alzheimer disease and other neurodegenerative disorders. Nat. Rev. Neurol..

[25] Gómez-Nicola D., Fransen N.L., Suzzi S. (2013). Regulation of microglial proliferation during chronic neurodegeneration. J. Neurosci..

[26] Yao J., Zhang M., Ma Q.-Y. (2011). PAd-shRNA-PTN reduces pleiotrophin of pancreatic cancer cells and inhibits neurite outgrowth of DRG. World J. Gastroenterol..

[27] Lee I.S., Jung K., Kim I.S. (2015). Human neural stem cells alleviate Alzheimer-like pathology in a mouse model. Mol. Neurodegener..

[28] Rangasamy S.B., Jana M., Roy A. (2018). Selective disruption of TLR2-MyD88 interaction inhibits inflammation and attenuates Alzheimer’s pathology. J. Clin. Invest..

[29] Tsai S.J. (2017). Effects of interleukin-1beta polymorphisms on brain function and behavior in healthy and psychiatric disease conditions. Cytokine Growth Factor Rev..

[30] Pons V., Lévesque P., Plante M.M. (2021). Conditional genetic deletion of CSF1 receptor in microglia ameliorates the physiopathology of Alzheimer’s disease. Alzheimers Res. Ther..

[31] Piehl N., van Olst L., Ramakrishnan A. (2022). Cerebrospinal fluid immune dysregulation during healthy brain aging and cognitive impairment. Cell.

[32] Pyonteck S.M., Akkari L., Schuhmacher A.J. (2013). CSF-1R inhibition alters macrophage polarization and blocks glioma progression. Nat. Med..

[33] Lawson L.J., Perry V.H., Dri P. (1990). Heterogeneity in the distribution and morphology of microglia in the normal adult mouse brain. Neuroscience.

[34] Bruttger J., Karram K., Wörtge S. (2015). Genetic cell ablation reveals clusters of local self-renewing microglia in the mammalian central nervous system. Immunity.

[35] Paolicelli R.C., Bolasco G., Pagani F. (2011). Synaptic pruning by microglia is necessary for normal brain development. Science.

[36] Parkhurst C.N., Yang G., Ninan I. (2013). Microglia promote learning-dependent synapse formation through brain-derived neurotrophic factor. Cell.

[37] Colonna M., Butovsky O. (2017). Microglia function in the central nervous system during health and neurodegeneration. Annu. Rev. Immunol..

[38] Serrano-Pozo A., Mielke M.L., Gómez-Isla T. (2011). Reactive glia not only associates with plaques but also parallels tangles in Alzheimer’s disease. Am. J. Pathol..

[39] Zhang H., Wei W., Zhao M. (2021). Interaction between Aβ and tau in the pathogenesis of Alzheimer’s disease. Int. J. Biol. Sci..

[40] Guttikonda S.R., Sikkema L., Tchieu J. (2021). Fully defined human pluripotent stem cell-derived microglia and tri-culture system model C3 production in Alzheimer’s disease. Nat. Neurosci..

[41] Ransohoff R.M. (2016). How neuroinflammation contributes to neurodegeneration. Science.

[42] Chen X., Firulyova M., Manis M. (2023). Microglia-mediated T cell infiltration drives neurodegeneration in tauopathy. Nature.

[43] Hopp S.C., Lin Y., Oakley D. (2018). The role of microglia in processing and spreading of bioactive tau seeds in Alzheimer’s disease. J. Neuroinflammation.

[44] Bhaskar K., Konerth M., Kokiko-Cochran O.N. (2010). Regulation of tau pathology by the microglial fractalkine receptor. Neuron.

[45] Taddei R.N., Sanchez-Mico M.V., Bonnar O. (2022). Changes in glial cell phenotypes precede overt neurofibrillary tangle formation, correlate with markers of cortical cell damage, and predict cognitive status of individuals at Braak III−IV stages. Acta Neuropathol. Commun..

[46] Krstic D., Madhusudan A., Doehner J. (2012). Systemic immune challenges trigger and drive Alzheimer-like neuropathology in mice. J. Neuroinflammation.

[47] Davies D.S., Ma J., Jegathees T. (2017). Microglia show altered morphology and reduced arborization in human brain during aging and Alzheimer’s disease. Brain Pathol..

[48] DiSabato D.J., Quan N., Godbout J.P. (2016). Neuroinflammation: The devil is in the details. J. Neurochem..

[49] McConnell H.L., Kersch C.N., Woltjer R.L. (2017). The translational significance of the neurovascular unit. J. Biol. Chem..

[50] Y.-M. Qiu, C.-L. Zhang, A.-Q. Chen, et al., Immune cells in the BBB disruption after acute ischemic stroke: Targets for immune therapy? Front. Immunol. 12 (2021), 678744. 10.3389/fimmu.2021.678744 PMC8260997 34248961

[51] Jolivel V., Bicker F., Binamé F. (2015). Perivascular microglia promote blood vessel disintegration in the ischemic penumbra. Acta Neuropathol..

[52] Wallet C., De Rovere M., Van Assche J. (2019). Microglial Cells: The main HIV-1 reservoir in the brain. Front. Cell. Infect. Microbiol..

[53] P. Chauhan, S. Hu, W.S. Sheng, et al., Regulatory T-cells suppress cytotoxic T lymphocyte responses against microglia, Cells 11 (2022), 2826. 10.3390/cells11182826 PMC9496959 36139401

[54] Goddery E.N., Fain C.E., Lipovsky C.G. (2021). Microglia and perivascular macrophages act as antigen presenting cells to promote CD8 T cell infiltration of the brain. Front Immunol..

[55] Tsai M.S., Wang L.C., Tsai H.Y. (2021). Microglia reduce Herpes simplex virus 1 lethality of mice with decreased T cell and interferon responses in brains. Int. J. Mol. Sci..

[56] Dulken B.W., Buckley M.T., Navarro Negredo P. (2019). Single-cell analysis reveals T cell infiltration in old neurogenic niches. Nature.

[57] Yan J., Xu W., Lenahan C. (2021). CCR5 activation promotes NLRP1-dependent neuronal pyroptosis via CCR5/PKA/CREB pathway after intracerebral hemorrhage. Stroke.

[58] P. Chauhan, W.S. Sheng, S. Hu, et al., Differential cytokine-induced responses of polarized microglia, Brain Sci. 11 (2021), 1482. 10.3390/brainsci11111482 PMC8615503 34827481

[59] Hefter D., Ludewig S., Draguhn A. (2020). Amyloid, APP, and electrical activity of the brain. Neuroscientist.

[60] Matza D., Kerem A., Shachar I. (2003). Invariant chain, a chain of command. Trends Immunol..

[61] Matsuda S., Matsuda Y., D’Adamio L. (2009). CD74 interacts with APP and suppresses the production of Aβ. Mol. Neurodegener..

[62] Kiyota T., Zhang G., Morrison C.M. (2015). AAV2/1 CD74 gene transfer reduces β-amyloidosis and improves learning and memory in a mouse model of Alzheimer’s disease. Mol. Ther..

[63] Erblich B., Zhu L., Etgen A.M. (2011). Absence of colony stimulation factor-1 receptor results in loss of microglia, disrupted brain development and olfactory deficits. PLoS One.

[64] Liu Y.-J., Spangenberg E.E., Tang B. (2021). Microglia elimination increases neural circuit connectivity and activity in adult mouse cortex. J. Neurosci..

[65] Feng X., Valdearcos M., Uchida Y. (2017). Microglia mediate postoperative hippocampal inflammation and cognitive decline in mice. JCI Insight.

[66] Wu S., Xue R., Hassan S. (2018). Il34-Csf1r pathway regulates the migration and colonization of microglial precursors. Dev. Cell.

[67] Suh H.S., Zhao M.-L., Derico L. (2013). Insulin-like growth factor 1 and 2 (IGF1, IGF2) expression in human microglia: Differential regulation by inflammatory mediators. J. Neuroinflammation.

[68] Carson M.J., Behringer R.R., Brinster R.L. (1993). Insulin-like growth factor I increases brain growth and central nervous system myelination in transgenic mice. Neuron.

[69] Cao Z., Harvey S.S., Chiang T. (2021). Unique subtype of microglia in degenerative thalamus after cortical stroke. Stroke.

[70] Shen X., Qiu Y., Wight A.E. (2022). Definition of a mouse microglial subset that regulates neuronal development and proinflammatory responses in the brain. Proc. Natl. Acad. Sci. U S A.

[71] Krasemann S., Madore C., Cialic R. (2017). The TREM2-APOE pathway drives the transcriptional phenotype of dysfunctional microglia in neurodegenerative diseases. Immunity.

[72] Hou L., Voit R.A., Sankaran V.G. (2020). CD11c regulates hematopoietic stem and progenitor cells under stress. Blood Adv..

[73] Helft J., Böttcher J., Chakravarty P. (2015). GM-CSF mouse bone marrow cultures comprise a heterogeneous population of CD11c^+^MHCII^+^ macrophages and dendritic cells. Immunity.

[74] Anderson S.R., Roberts J.M., Zhang J. (2019). Developmental apoptosis promotes a disease-related gene signature and independence from CSF1R signaling in retinal microglia. Cell Rep..

[75] Kohno K., Shirasaka R., Yoshihara K. (2022). A spinal microglia population involved in remitting and relapsing neuropathic pain. Science.

[76] Shi Y., Holtzman D.M. (2018). Interplay between innate immunity and Alzheimer disease: APOE and TREM2 in the spotlight. Nat. Rev. Immunol..

[77] Rangaraju S., Raza S.A., Li N.X. (2018). Differential phagocytic properties of CD45^low^ Microglia and CD45^high^ brain mononuclear phagocytes-activation and age-related effects. Front. Immunol..

[78] Goudarzi S., Gilchrist S.E., Hafizi S. (2020). Gas6 induces myelination through anti-inflammatory IL-10 and TGF-β upregulation in white matter and glia. Cells.

[79] Huang Y., Happonen K.E., Burrola P.G. (2021). Microglia use TAM receptors to detect and engulf amyloid β plaques. Nat. Immunol..

[80] Fourgeaud L., Través P.G., Tufail Y. (2016). TAM receptors regulate multiple features of microglial physiology. Nature.

[81] J.Y. Kim, J. Kim, M. Huang, et al., CCR4 and CCR5 involvement in monocyte-derived macrophage migration in neuroinflammation, Front. Immunol. 13 (2022), 876033. 10.3389/fimmu.2022.876033 PMC9133420 35634277

[82] Haruwaka K., Ikegami A., Tachibana Y. (2019). Dual microglia effects on blood brain barrier permeability induced by systemic inflammation. Nat. Commun..

[83] Joy M.T., Ben Assayag E., Shabashov-Stone D. (2019). CCR5 is a therapeutic target for recovery after stroke and traumatic brain injury. Cell.

[84] Maudet C., Kheloufi M., Levallois S. (2022). Bacterial inhibition of Fas-mediated killing promotes neuroinvasion and persistence. Nature.

[85] Mizutani M., Pino P.A., Saederup N. (2012). The fractalkine receptor but not CCR2 is present on microglia from embryonic development throughout adulthood. J. Immunol..

[86] Semple B.D., Bye N., Rancan M. (2010). Role of CCL2 (MCP-1) in traumatic brain injury (TBI): Evidence from severe TBI patients and CCL2-/- mice. J. Cereb. Blood Flow Metab..

[87] Hsieh C.L., Niemi E.C., Wang S.H. (2014). CCR2 deficiency impairs macrophage infiltration and improves cognitive function after traumatic brain injury. J. Neurotrauma.

[88] Somebang K., Rudolph J., Imhof I. (2021). CCR2 deficiency alters activation of microglia subsets in traumatic brain injury. Cell Rep..

[89] Spittau B., Dokalis N., Prinz M. (2020). The role of TGFβ signaling in microglia maturation and activation. Trends Immunol..

[90] Yoshinaga K., Obata H., Jurukovski V. (2008). Perturbation of transforming growth factor (TGF)-β1 association with latent TGF-β binding protein yields inflammation and tumors. Proc. Natl Acad. Sci. U S A.

[91] Brionne T.C., Tesseur I., Masliah E. (2003). Loss of TGF-β1 leads to increased neuronal cell death and microgliosis in mouse brain. Neuron.

[92] Hickman S.E., Kingery N.D., Ohsumi T.K. (2013). The microglial sensome revealed by direct RNA sequencing. Nat. Neurosci..

[93] Buttgereit A., Lelios I., Yu X. (2016). Sall1 is a transcriptional regulator defining microglia identity and function. Nat. Immunol..

[94] Zöller T., Schneider A., Kleimeyer C. (2018). Silencing of TGFβ signalling in microglia results in impaired homeostasis. Nat. Commun..

