# Supplementary material for: The role of signaling crosstalk of microglia in hippocampus on progression of ageing and Alzheimer's disease
Source: J Pharm Anal. 2023 May 15;13(7):788–805. doi: 10.1016/j.jpha.2023.05.008 (PMC10422165; doi:10.1016/j.jpha.2023.05.008)
Supplement: Multimedia component 3 [file mmc3.docx]

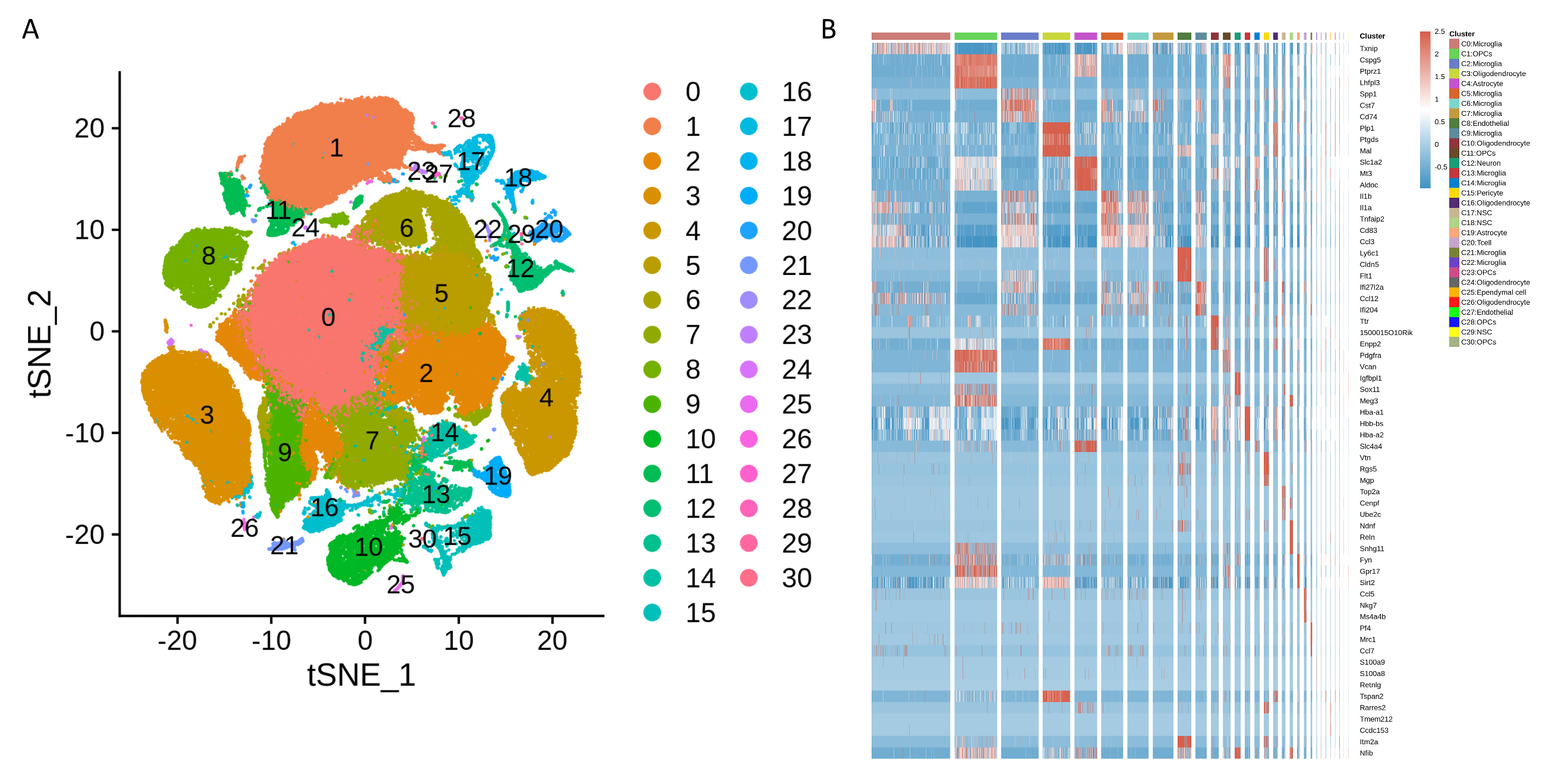


Fig. S1. (A). t-Stochastic Neighbor Embedding (t-SNE) dimensional reduction. (B). Heatmap presenting Top 3 genes from each cluster.


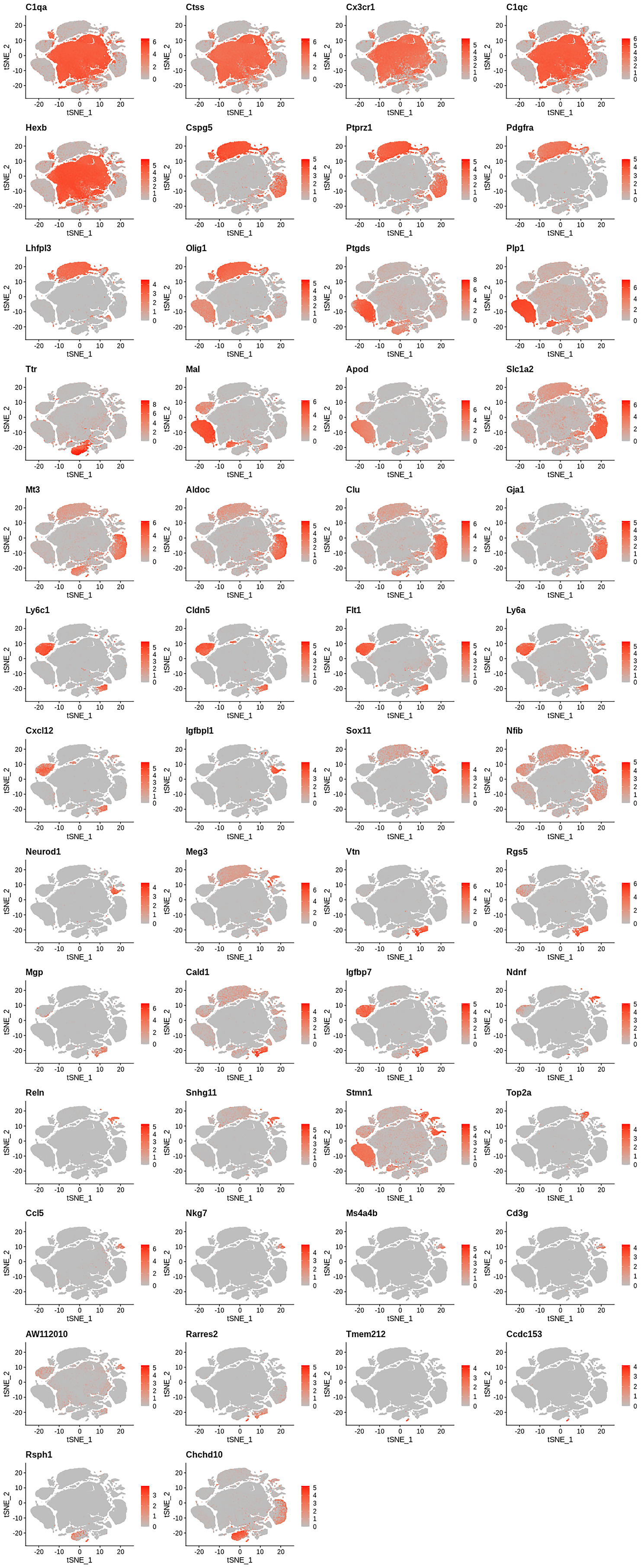


Fig. S2. Uniform manifold approximation and projection reveal marker genes distribution.


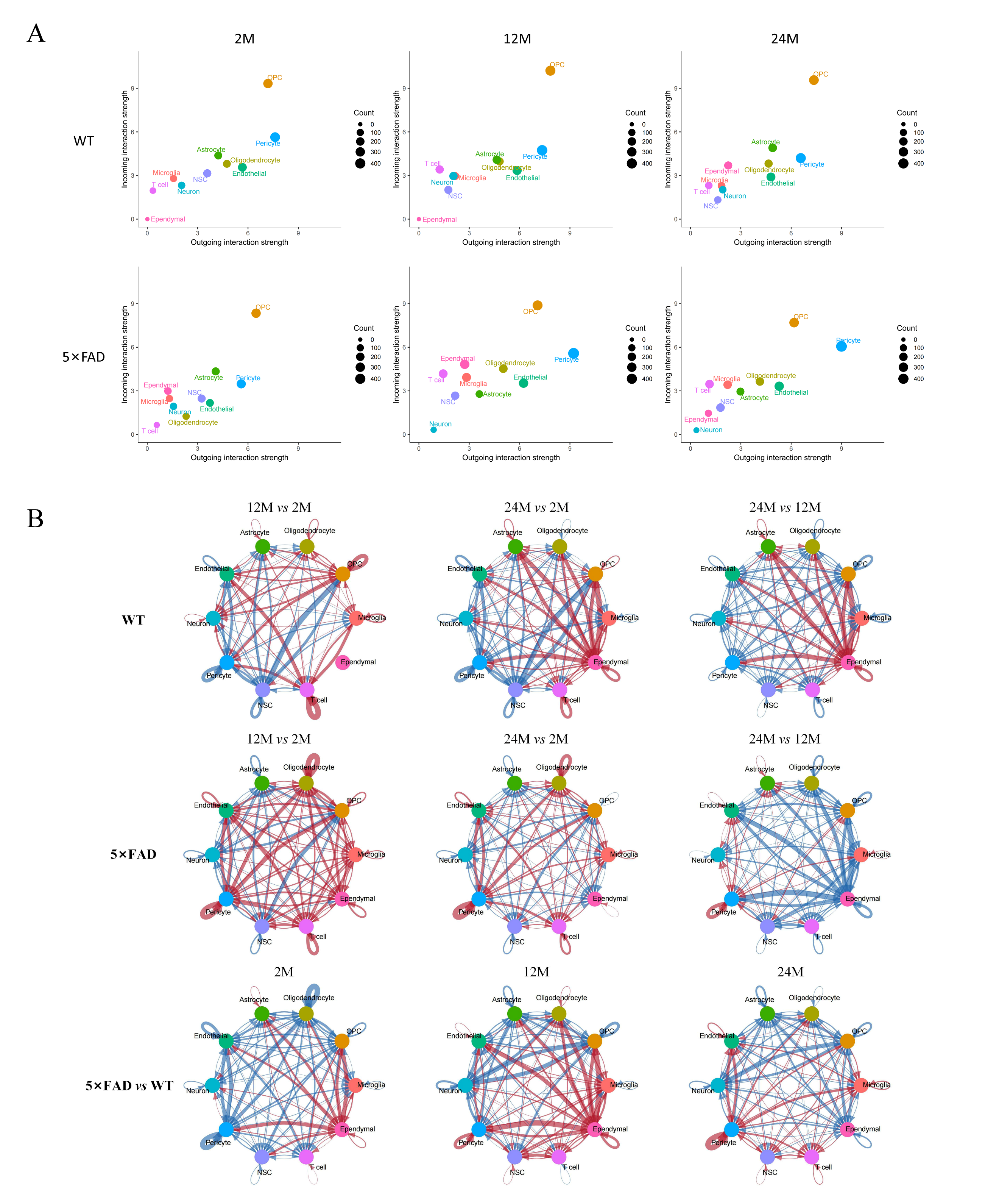


Fig. S3. (A) The bubble diagram shows the outgoing and incoming interaction strength comparison of cell types in the 2-, 12-, and 24-month-old wild type (WT) and 5×Familiar Alzheimer Disease (5×FAD) groups. (B). Signal strength was changed. OPCs: oligodendrocyte progenitor cells; NSC: neural stem cells.


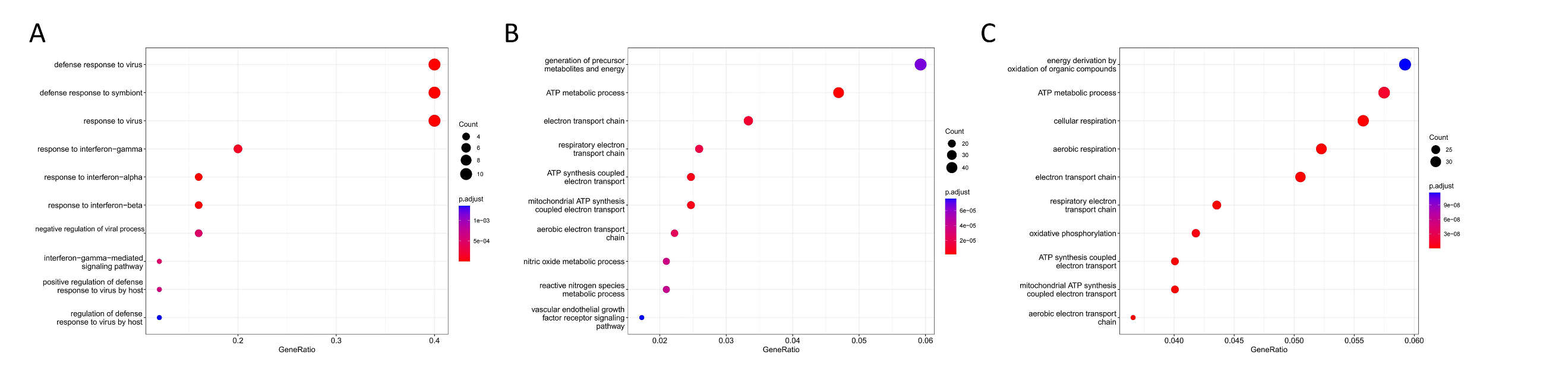


Fig. S4. Gene Ontology analysis annotated the biological process of Alzheimer Disease process.

(A) At 2- month age, 5×Familiar Alzheimer Disease (5×FAD) *vs* wild type (WT). (B) At 12- month age, 5×FAD *vs* WT. (C) At 24- month age, 5×FAD *vs* WT.


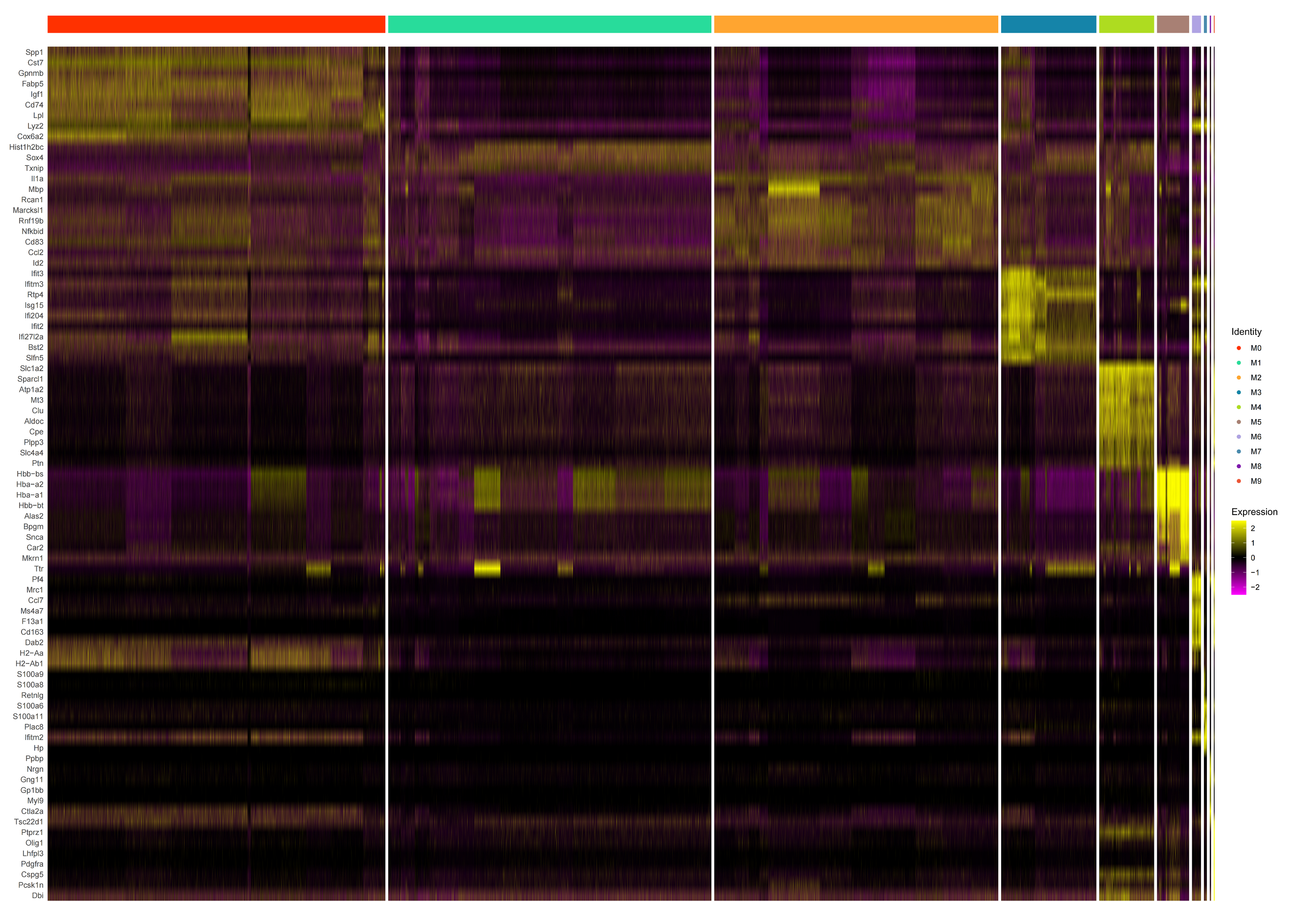


Fig. S5. Marker genes for each microglial subtype.


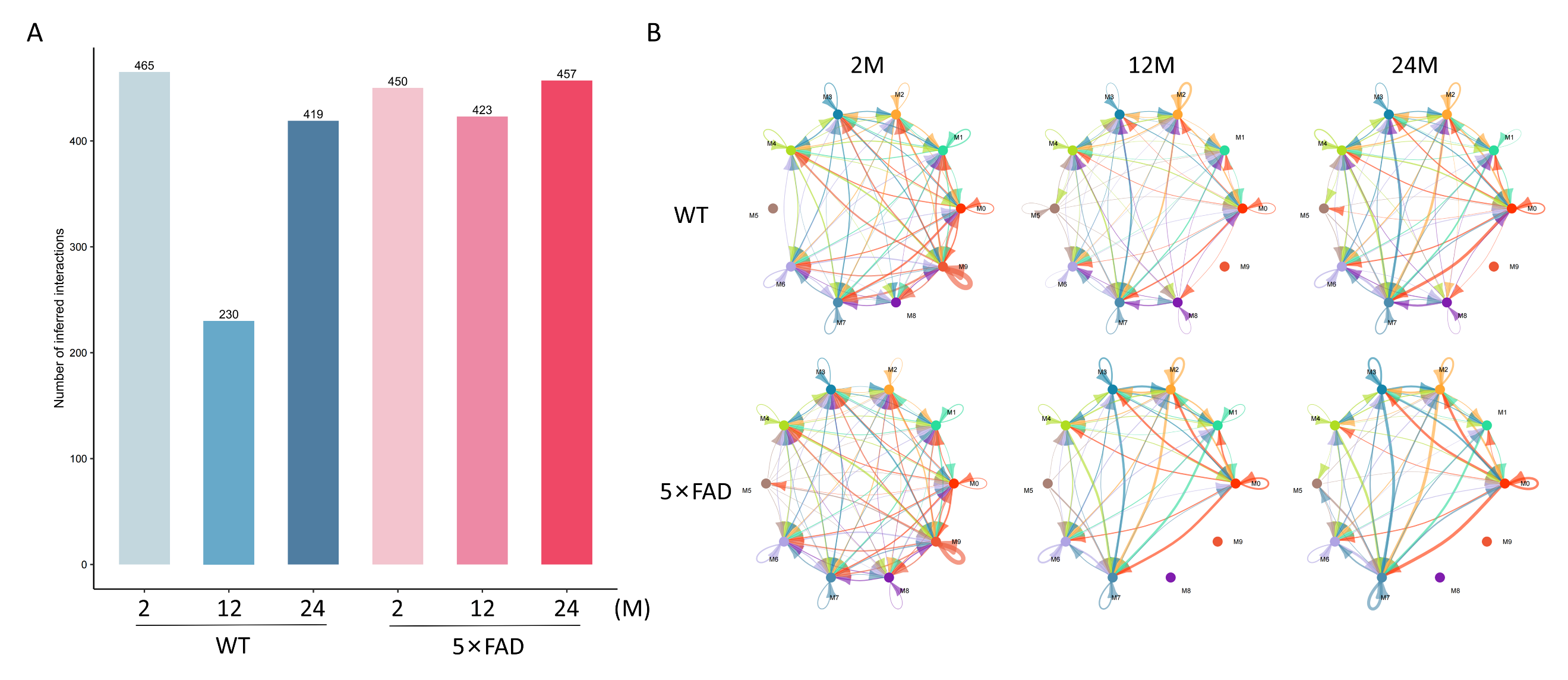


Fig. S6. (A) Total number of interactions from microglia. (B) The number of interactions between microglia subtypes.
